# Structural insights into plant phytochrome A as a highly sensitized photoreceptor

**DOI:** 10.1038/s41422-023-00858-4

**Published:** 2023-07-25

**Authors:** Yuxuan Zhang, Xiaoli Lin, Chengying Ma, Jun Zhao, Xiaojin Shang, Zhengdong Wang, Bin Xu, Ning Gao, Xing Wang Deng, Jizong Wang

**Affiliations:** 1 National Key Laboratory of Wheat Improvement, Peking University Institute of Advanced Agricultural Sciences, Shandong Laboratory of Advanced Agriculture Sciences at Weifang, Weifang, Shandong China; 2 State Key Laboratory of Protein and Plant Gene Research, School of Advanced Agricultural Sciences, Peking University, Beijing, China; 3 Peking-Tsinghua Joint Center for Life Sciences, Peking University, Beijing, China; 4 State Key Laboratory of Membrane Biology, School of Life Sciences, Peking University, Beijing, China

**Keywords:** Electron microscopy, Plant signalling

Dear Editor,

Phytochromes (phys), first discovered in plants, are red and far-red photoreceptors that are also widely found in bacteria and fungi later.^1,2^ Plant phys utilize the linear tetrapyrrole chromophore called photochromobilin (PΦB). Phys reversibly toggle between the red light-absorbing Pr and the far-red light-absorbing Pfr conformers by photoconversion, with absorption peaks at ~667 nm and ~730 nm, respectively. The Pfr conformer can be converted to Pr in darkness, a process called dark reversion or thermoreversion.^2,3^ In plants, Pr conformers reside in the cytoplasm; and upon photoconversion, Pfr conformers are translocated into the nucleus, resulting in a plethora of physiological and developmental changes throughout the plant life cycle.

The higher plant *Arabidopsis thaliana* encodes five *phy* genes, designated *phyA*–*E*.^4^ PhyB is the major red light receptor mediating the classical red/far-red light reversible low fluence response (LFR) or red light high irradiance response (R-HIR). PhyC–E mostly perform a complementary function to that of phyB in adult plants.^2,3^ PhyA is distinct from other phys and is responsible for the very low fluence response (VLFR) under a broad spectrum of light and for the far-red light high irradiance response (FR-HIR).^2,3^ Thus, phyA has higher photosensitivity than phyB and is intrinsically more sensitive to light.^5,6^

In higher plants, phys share a conserved domain structure consisting of an N-terminal photosensory module (PSM) followed by two tandem Period/ARNT/Singleminded (PAS) domains, and a C-terminal histidine kinase-related domain (HKRD).^1,2^ The PSM comprises four consecutive domains: N-terminal extension (NTE), N-terminal PAS (nPAS), cGMP phosphodiesterase/adenylate cyclase/FhlA (GAF) and phytochrome-specific (PHY) domains. The HKRD can be further divided into dimerization histidine phosphotransfer (DHp) and catalytic ATP-binding (CA) subdomains. Although several phyB-related structures have been reported,^7–9^ the structure of full-length phyA is still lacking, hindering the characterization of structural differences between phyA and phyB.

To solve the structure of phyA, we expressed full-length dicot *A. thaliana* phyA (AtphyA) and monocot *Zea mays* phyA1 (ZmphyA1) proteins in insect cells and PΦB chromophore in *Escherichia coli* cells. Size-exclusion chromatography assays demonstrated that PΦB-free phyA (apo-phyA) and Pr conformer of phyA (phyA-Pr) have a similar molecular weight of ~250 kDa (Supplementary information, Fig. S1a, b). The absorption peak of the phyA-Pr protein is 665 nm but shifts to 725 nm following saturating irradiation with red light (Supplementary information, Fig. S1c, d). Together with the cyan color of phyA protein solution and a corroborating zinc-induced fluorescence assay (Supplementary information, Fig. S1), these results confirmed that the phyA proteins were correctly assembled.

Then, we solved cryo-electron microscopy (cryo-EM) structures of apo-AtphyA, AtphyA-Pr and ZmphyA1-Pr at resolutions of 3.8 Å, 3.0 Å and 3.3 Å, respectively (Supplementary information, Figs. S2–S4 and Table S1). The three proteins form similar homodimers with conserved topology (Fig. 1a, b; Supplementary information, Figs. S5, S6). In the homodimeric structures, “head-to-tail” packing of the two PSM–PAS2 modules forms a parallelogram-shaped platform, occupying a surface area of ~ 9000 Å^2^. The homodimeric HKRD packing “head-to-head” protrudes from the PSM–PAS2 platform and tilts slightly to one side of the platform (Fig. 1b; Supplementary information, Fig. S5). In all the reconstructed cryo-EM maps, most parts of the NTE and the entire PAS1 were not well-defined (Supplementary information, Table S2). Interestingly, the PAS1 domains of AtphyA-Pr and ZmphyA1-Pr position differently in the low-pass filtered cryo-EM maps (Supplementary information, Fig. S5d). Furthermore, the residues between PHY and PAS1 vary substantially between the two phyAs and other phy species (Supplementary information, Fig. S7).

**Fig. 1:**
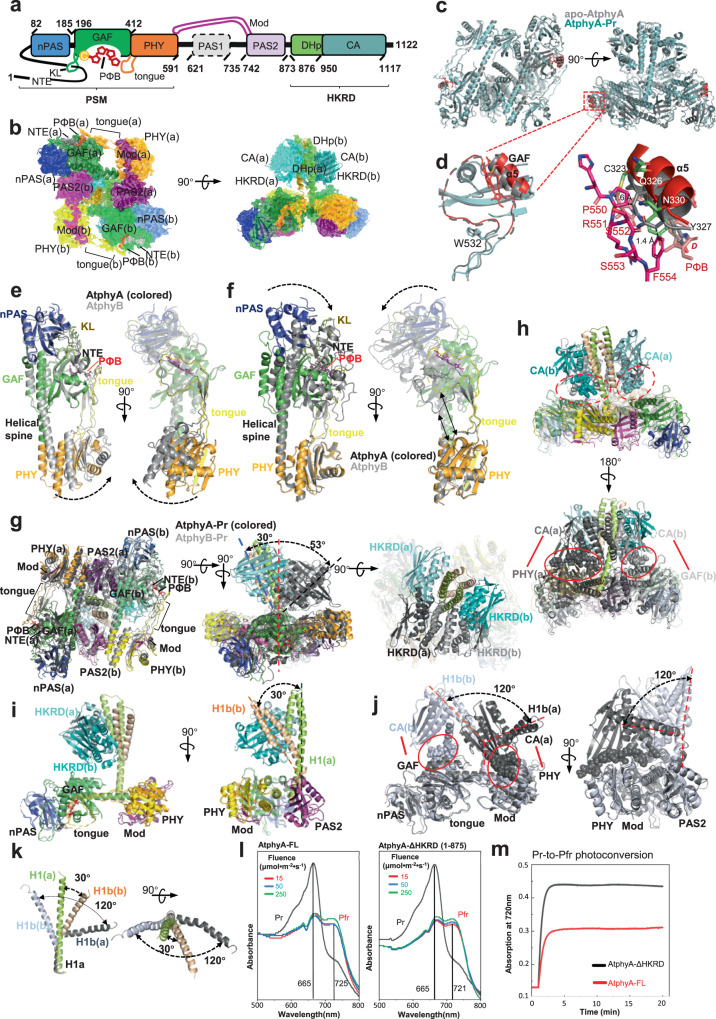
Structural features of the Pr conformer of plant phytochrome A contribute to its high photosensitivity. **a** Color-coded domain architecture of full-length AtphyA: the PSM containing NTE, nPAS, GAF and KL, PHY and tongue; the PAS1–PAS2 and Mod; the HKRD comprising DHp and CA subdomains. **b** Cryo-EM map of AtphyA-Pr superposed with an atomic model in cartoon shown in two orientations. **c** Structural comparison of AtphyA-Pr (cyan) and apo-AtphyA (gray) shown in two orientations. **d** Left: the differences between AtphyA-Pr (red) and apo-AtphyA (gray) in the GAF helix-α5 and the PHY tongue. Right: detailed interactions between PRSSF motif and GAF helix-α5. The numbers in angstroms indicate the prohibitively close distances between residues in apo-AtphyA helix-α5 and PRSSF motif. **e** Structural comparison of PSMs of AtphyA-Pr (color-coded) and AtphyB-Pr (gray, PDB code: 7RZW) using nPAS–GAF as the template shown in two orientations. The dashed arrows indicate the staggered PHY domains. **f** Structural comparison in **e** using PHY as the template shown in two orientations. The dashed arrows indicate the staggered nPAS–GAF modules. The double arrows indicate the two PHY tongues with different curvatures. **g** Structural comparison of AtphyA-Pr (color-coded) and AtphyB-Pr (gray, PDB code: 7RZW) shown in three orientations. The tilting angles of the HKRD dimers quantified as angles between the central axis of the PSM–PAS2 platform (dashed red line) and the pseudo-C2 axis (dashed cyan line for phyA and dashed black line for phyB) of HKRD dimers are indicated. **h** Structural comparison in **g** highlighting the packing of HKRDs against PSMs. The dashed red ellipses highlight residues (in spheres) of the CA subdomains close to the PSMs in AtphyA-Pr. The red ellipses delineate residues (in spheres) of the CA subdomains interacting with the PHY and the GAF domains, respectively, in AtphyB-Pr. The red lines indicate the two contacting domains. **i** Structural comparison of the two protomers in AtphyA-Pr shown in two orientations. **j** Structural comparison of the two protomers in AtphyB-Pr (PDB code: 7RZW) shown in two orientations. The contacting residues are shown in spheres and delineated by red ellipses. **k** Structural alignment of four H1 helices in AtphyA-Pr (color-coded) and AtphyB-Pr (gray, PDB code: 7RZW) shown in two orientations. **l** The absorption spectra of full-length AtphyA and HKRD-truncated AtphyA were recorded after irradiating Pr for 20 min with increasing fluences of 665-nm light. **m** Simulated kinetic profiles for Pr-to-Pfr photoconversion as monitored by the gain in Pfr absorption at 720 nm.

The PSMs of AtphyA-Pr and ZmphyA1-Pr are structurally identical, as observed in the crystal structure of the NTE–nPAS–GAF fragment of *Glycine max* phyA^8^ (Supplementary information, Fig. S8a–c). Specifically, we well defined a small NTE fragment, the knot lasso (KL) in the GAF domain, the tongue protrusion in the PHY domain and the PΦB molecule adopting a 5(*Z*)*syn*-10(*Z*)*syn*-15(*Z*)*anti* configuration in phyA-Pr (Supplementary information, Fig. S8d–f). In contrast, the 150s loop (T108–V122) in the nPAS domain and the 380s loop (N344–K361) in the GAF domain are completely disordered (Supplementary information, Fig. S8a), as in phyB-Pr.^7–9^ The crucial residues of phyA-Pr involved in PΦB binding are conserved in other phys across species (Supplementary information, Figs. S7, S8g), further confirming the conserved mechanisms of PΦB assembly in plant phys.^7–11^

Structural comparison of apo-AtphyA and AtphyA-Pr revealed that they share a highly similar homodimeric structure (Fig. 1c). However, notable differences were found around the PΦB-binding cradle. Except for those in the GAF helix-α5, the key residues involved in PΦB binding retain similar conformations in apo and Pr states of phyA (Supplementary information, Fig. S9). Tyr327 of apo-phyA helix-α5 can cause obvious steric clash with the D ring of PΦB. Thus, helix-α5 of AtphyA-Pr moves outwards (Fig. 1d), to accommodate the bulk of PΦB and facilitate the formation of covalent thioether linkage between them. However, the precise mechanism underlying this autoassembly reaction needs further investigation. Another intriguing question is how PΦB might gain access to its binding cradle. Previous studies indicate that WGG and PRSSF motifs in the PHY tongue contact the PΦB-binding cradle; and in plant phys, the open structure between these two motifs presumably forms an entrance for PΦB to its binding pocket.^7–9,12,13^ Similarly, in AtphyA-Pr, the PRSSF motif contacts GAF helix-α5 through hydrophobic interaction with its Phe554. Nonetheless, this contact is probably abolished in apo-AtphyA, as a closer location of GAF helix-α5 might cause substantial steric clashes with the PRSSF motif (Fig. 1d). Thus, the portion downstream of the WGG motif in the PHY tongue of apo-phyA becomes more disordered than that of phyA-Pr, facilitating the entrance of PΦB to its pocket. It is noteworthy that the tip of the PHY tongue in ZmphyA1-Pr also seems more flexible, as in apo-AtphyA. Considering the good alignment of GAF helix-α5 with AtphyA-Pr together with the less well-defined EM map densities of PΦB as well as the abnormal absorbance property of Pr conformers (Supplementary information, Figs. S1d, S8a, b, f), we speculate that protein sample quality might impinge on the EM map reconstruction of a complete tongue of ZmphyA1-Pr. Further investigations are needed to address this issue.

Structural alignment of the PSMs in AtphyA-Pr and AtphyB-Pr revealed that the two NTE–nPAS–GAF modules share similar structures (Fig. 1e). However, the PHY domains become staggered except for the tongue region that contacts the GAF domain (Fig. 1e). Notably, the tongue in AtphyA-Pr adopts a relatively stretched conformation with its tip locating much farther from the bulk of PHY. In contrast, a more curved tongue is found in AtphyB-Pr (Fig. 1f). As the tongues of both phyA and phyB exclusively contact the surface between the GAF and PHY domains (Fig. 1e, f), this structural divergence likely gives rise to the different arrangements of PHY in AtphyA-Pr and AtphyB-Pr.

The C-terminal region of phyA mediates its dimerization. The modulator loop (Mod) of PAS2 organizes the intraprotomer interface as in AtphyB-Pr,^9^ but the residues participating in the PAS2–(nPAS–GAF) interprotomer interface are not well-conserved between the two phys (Supplementary information, Fig. S10a–d). The PAS2-mediated dimerization interfaces are highly conserved between AtphyA-Pr and ZmphyA1-Pr (Supplementary information, Fig. S10e, f). Although the resolution of phyA HKRDs, especially the β-sheets in the CA subdomains, is not high enough to allow us to analyze side-chain interactions (Supplementary information, Figs. S2–S4 and Table S2), the H1 and H2 helices in DHp subdomains as well as α1, α2, α4 in CA subdomains are sufficiently well-defined in the cryo-EM map (Supplementary information, Fig. S10g). The phyA-Pr HKRD dimer is essentially asymmetric due to a striking kink that occurs only at H1 of protomer b, resulting in local disruption of the symmetry (Supplementary information, Fig. S10h). Structural comparison revealed marked divergences in the orientation of H1a between AtphyA-Pr and AtphyB-Pr. Both H1 helices of AtphyB-Pr possess kinks, resulting in both H1a helices oriented distinctly from those of AtphyA-Pr (Supplementary information, Fig. S10i).

Structural comparison of AtphyA-Pr and AtphyB-Pr revealed that the symmetric PSM–PAS2 parallelograms are well-superposed, although the AtphyA-Pr dimer occupies a slightly larger area than that of AtphyB-Pr (~8000 Å^2^) (Fig. 1g). This is consistent with the more stretched-out AtphyA-Pr PSM (Fig. 1e, f). The most striking difference between AtphyA-Pr and AtphyB-Pr occurs in the HKRD dimer tilt angles relative to the PSM–PAS2 platform, which are ~30° and ~53°, respectively (Fig. 1g). The larger tilt angle of AtphyB-Pr results in a more extensive contact between the HKRD and PSM. In contrast, because of a smaller tilt angle in AtphyA-Pr, only marginal interactions are established between these two structural domains (Fig. 1h). This is also seen in ZmphyA1-Pr, which has a tilt angle comparable to that of AtphyA-Pr (Supplementary information, Fig. S11a, b).

Structural comparison of the two protomers of AtphyA-Pr revealed an asymmetric dimer (Fig. 1i) resembling AtphyB-Pr,^9^ but there are notable differences in the packing of HKRDs against PSMs. The angle between the two H1 helices in AtphyA-Pr is ~30°, and there are no obvious intraprotomer contacts between HKRD–CA and PSM in either phyA-Pr protomer (Fig. 1i); this angle in AtphyB-Pr is ~120°, which is much larger than an ~30° angle in AtphyA-Pr, and intraprotomer interactions are established between the HKRD–CA and the PHY domain in one protomer and the GAF domain in the other protomer (Fig. 1j). In AtphyA-Pr and AtphyB-Pr, the four H1 helices in the HKRDs are organized differently owing to their kinks (Fig. 1k). Sequence alignment revealed that the amino acids of the kink region are not conserved among phy members (Supplementary information, Fig. S11c).

Previous studies established that phyA is a highly sensitized photoreceptor mediating the physiological activities of VLFR.^5,6^ Quantification assays showed that AtphyA is ~100-fold more sensitive to red light than AtphyB to saturate photoconversion from Pr to Pfr.^14^ An HKRD-deleted version of AtphyB effectively improves its photosensitivity to a level comparable to AtphyA.^15^ For AtphyA, the proteins of its HKRD-deleted mutant (AtphyA-ΔHKRD, 1–875 aa) purified by size exclusion chromatography are a mixture of homodimers and monomers (Supplementary information, Fig. S12a), consistent with the structural observation that both PAS2 and HKRD are involved in the dimerization of phyA-Pr. Furthermore, both the full length AtphyA-Pr (AtphyA-FL) and AtphyA-ΔHKRD can effectively sense red light with a gradient of low fluence rate (Fig. 1l), indicating their similar photosensitivity. However, simulated kinetics for Pr-to-Pfr photoconversion demonstrated a higher proportion of Pfr conformers and a faster rate of photoconversion for AtphyA-ΔHKRD (Fig. 1m; Supplementary information, Fig. S12b–d). Collectively, these results indicate that HKRD plays a role in restraining the photosensitivity of plant phys.

In conclusion, our structural analysis provides a mechanistic explanation for why phyA is a highly sensitized photoreceptor. Phy proteins exist in an equilibrium between Pr and Pfr states. Thus, the conformational stability of phy proteins might play a role in shifting this equilibrium. Structural comparison revealed much more extensive HKRD–PSM contacts in AtphyB-Pr than in AtphyA-Pr, indicating its higher conformational stability. The lower stability of AtpyA-Pr is expected to be more favorable for Pr-to-Pfr photoconversion. This may contribute to the higher photosensitivity of AtphyA. In addition, the thermal reversion rate of AtphyA is ~18-fold lower than that of AtphyB,^14^ indicating a more stable conformation of AtphyA-Pfr compared to AtphyB-Pfr. Whether the differences in PSM conformation, PSM–PAS2 interaction and HKRD–PSM interaction in AtphyA-Pr contribute to its Pfr stability remains to be investigated. Clearly, structures of phy proteins in their Pfr conformers will provide further mechanistic insights into their spectral characteristics. It should be mentioned that in addition to conformational stability, abundance, subcellular localization and half-life of phy proteins are also important in this aspect.

### Supplementary information

phyA-Pr_Supplementary information

## Data Availability

For apo-AtphyA, AtphyA-Pr, ZmphyA1-Pr, the atomic coordinates have been deposited in the Protein Data Bank with accession codes 8ISI, 8ISJ and 8ISK, respectively, and the EM maps have been deposited in the Electron Microscopy Database with accession codes EMD-35691, EMD-35692 and EMD-35693, respectively.
