# Supplementary material for: Structural insights into plant phytochrome A as a highly sensitized photoreceptor
Source: Cell Res. 2023 Jul 25;33(10):806–9. doi: 10.1038/s41422-023-00858-4 (PMC10542756; doi:10.1038/s41422-023-00858-4)
Supplement: Supplementary file 1 — phyA-Pr_Supplementary information [file 41422_2023_858_MOESM1_ESM.pdf]

MATERIALS AND METHODS

Expression and purification of phyA

The constructs of full-length AtphyA (residues 1–1127) and full length ZmphyA1 (1-1132, codon-optimized in GENEWIZ, Inc.) generated by standard PCR-based cloning strategy were cloned into the pFastBac-1 vector (Invitrogen) with bearing an N-terminal 6×His-SUMO tag. The *Synechocystis* PCC6803 haem oxygenase (Ho1) and *Arabidopsis thaliana* PΦB synthase (HY2) were cloned into the pRSFDuet-1 vector without any tags. Their identities were confirmed by sequencing. The apo-phyA proteins were expressed in High Five insect cells at 22 °C. One litre of cells (2.0×10^6 cells mL^{-1} cultured in the medium from Expression Systems) was infected with 20 ml recombinant baculovirus and the cells were harvested after 48 h. The PΦB-expressing BL21 (DE3) cells were grown in the dark at 37 °C in Terrific Broth containing 250 μM 5-aminolevulinic acid with continuous shaking to an OD_{600} of about 0.6. Then the culture temperature was changed to 16 °C, and 1 mM isopropyl β-D-1-thiogalactopyranoside (IPTG) was added to induce PΦB synthesis for 6 h. For the purification of phyA-Pr proteins, all the steps were conducted in darkness or under dim green light. One litre of the apo-phyA-expressing insect cells and 200 mL PΦB-expressing BL21 (DE3) cells were separately harvested at 4 °C by centrifugation at 4200×g for 15 min. Then the cell pellets containing the apo-proteins and PΦB were resuspended with Lysis Buffer (20 mM Tris-HCl (pH 8.0), 5 % (v/v) glycerol, 150 mM NaCl, 20 mM imidazole) and mixed together. Then the mixture was disrupted by sonication on ice. Following centrifugation at 25000×g for 1h, the phyA-Pr proteins were further purified using Ni-NTA (Novagen) from the supernatant. All the recovered proteins were then digested with PPase to remove the 6×His-SUMO tag and further purified by ion-exchange (Mono Q™ 10/100 GL, Cytiva) and gel-filtration (Superose™ 6 Increase 10/300 GL, Cytiva) in buffer containing 20 mM Tris-HCl pH 8.0 and 150 mM NaCl. The AtphyA HKRD-deleted mutant proteins were purified as described above. The apo-phyA proteins were directly purified from the High Five insect cells with similar procedure as described above.

Gel filtration assay

All the gel filtration (Superose™ 6 Increase 10/300 GL, Cytiva) assays were performed in buffer containing 20 mM Tris-HCl pH 8.0 and 150 mM NaCl. The assay was performed with a flow rate of 0.5 mL/min and an injection volume of 2 mL protein samples. Ovalbumin (44 kDa), aldolase

(158 kDa), ferritin (440 kDa) and thyroglobulin (669 kDa) were used as standard protein markers. The integrity of the final phyA preparations was confirmed by UV280 absorption spectra, and by SDS-PAGE followed by staining of the gels either for protein with Coomassie Brilliant Blue or for PΦB by zinc-induced fluorescence under UV light.

UV-Vis Spectroscopy and kinetic analyses

Absorption spectra of Pr-to-Pfr photoconversion in Fig. 1l and Fig. S1 were measured by UV-1900i spectrophotometer (Shimadzu) at 25 °C in the dark. To maintain the consistency of experimental results, the cuvette and sample volumes were kept constant for all measurements. To increase the proportion of the Pr state as much as possible, 1 mL of purified phyA proteins was treated with far-red light laser (740 nm, 50.41 $\mu mol \cdot m^{-2} \cdot s^{-1}$) irradiation for 20 min on ice before assay. Pr spectra were collected from 500-800 nm while the scan rate was set at maximum to minimize the red or far-red light exposure. Pr-to-Pfr photoconversion was driven with different fluence red light laser (660 nm, 15 $\mu mol \cdot m^{-2} \cdot s^{-1}$, 50 $\mu mol \cdot m^{-2} \cdot s^{-1}$, 118.8 $\mu mol \cdot m^{-2} \cdot s^{-1}$, 250 $\mu mol \cdot m^{-2} \cdot s^{-1}$) for 30 min on ice, and then collected Pfr spectra as above. The Pfr spectrum was subtracted from the Pr spectrum to generate difference spectra. Time-resolved Pr-to-Pfr photoconversion data in Fig. 1m and Fig. S12 were collected in red light irradiation by Cary 4000 UV-Vis Spectrophotometer (Agilent) after driving the Pr levels to steady state with a 740 nm LED. Pr-to-Pfr photoconversions were driven by LED with peak emissions of 670 nm and the fluence rate of 30 $\mu mol \cdot m^{-2} \cdot s^{-1}$. Simulated kinetic profiles for photoconversion as monitored by the gain in Pfr absorption at 720 nm to show the relative absorption changes for the samples.

Cryo-EM sample preparation and data collection

For cryo-EM sample preparation, 3.5 μL aliquot of apo-AtphyA, AtphyA-Pr, and ZmphyA1-Pr sample were applied to discharged 300-mesh Au R1.2/1.3 grids (C-flat). Grids were blotted for 2-3.5 s and plunged into liquid ethane using an FEI Mark IV Vitrobot operated at 4 °C and 100% humidity. Cryo-EM data were collected on a Titan Krios (ThermoFisher Scientific) electron microscope operated at 300 kV and a Gatan K3 Summit direct electron detection camera (Gatan) using EPU software. Micrographs were recorded in super-resolution mode at a nominal magnification of 105,000 ×, resulting in a physical pixel size of 0.85 Å per pixel. Defocus values

varied from -1 μm to -2 μm. The dose rate was 15 electron per pixel per second. Exposures of 2.41 s were dose-fractionated into 40 subframes, leading to a total accumulated dose of 50 electrons per Å^2.

Image processing and 3D reconstruction

For the apo-AtphyA, 8,117 movies were collected. Motion correction were performed using MotionCor2 (ref.1) and the contrast transfer function (CTF) parameters were estimated with the Gctf[2]. RELION[3] (versions 4.0) were used for particle picking, 2D and 3D classification and structural refinement. 6,300,392 autopicked particles were subjected to 2D classification, resulting in a dataset of 2,601,275 particles for further processing. After rounds of 2D and 3D classification, the particles were divided to three groups and subjected to 3D refinement. To explore the structural dynamics of PhyA, each group particles were subjected to 3D classification. The resulting classes with similar orientation were combined and subjected to further 3D refinement. As the flexibility of HK domain, masked 3D classification and refinement was used to obtain high resolution structure. A total of 873,384 particles were kept for final 3D refinement and model building (final resolution 3.77 Å). Parameters for data processing and 3D reconstruction are summarized in Figure S4 and Table S1.

For AtphyA-Pr, 10,864 movies were collected. The raw super-solution dose-fractionated images stacks were 2 × Fourier binned, aligned, dose-weighted and summed using Patch Motion Correction in cryoSPARC[4] (versions 4.1.2), resulting in summed micrographs in a pixel size of 0.85 Å per pixel. Contrast transfer function (CTF) parameters were estimated using Patch CTF Estimation in cryoSPARC. After excluding the micrographs with bad Thon rings, 10,457 micrographs were selected for subsequent processing. A total of 8,006,050 particles were auto-picked using cryoSPARC Template Picker. 2D templates used for particles auto-picking was generated by emd_24780. Then several rounds of reference-free 2D classification were performed to remove contaminants and noise in the raw automatically picked particles. The selected particles after 2D classification were subjected to ab-initio reconstruction requesting three classes in cryoSPRC. Then 3D classification was performed using the 3D map of the best class in the last step as the initial reference model. A total of 774,153 particles were selected for the final 3D auto-refinement. The AtphyA-Pr yielded a particle density with an estimated resolution of 3.03 Å for model building. Reconstruction resolutions were determined based on the gold-standard Fourier

shell correlation (FSC) 0.143 criterion with the high-resolution noise substitution. Parameters for data processing and 3D reconstruction are summarized in Figure S2 and Table S1.

For ZmphyA1-Pr, it was processed similarly to the AtPhyA-Pr dataset, as shown in Figure S3 and Table S1. 8,996 CTF-corrected cryo-EM images were manually selected and totally extracted 6,942,083 particles for 2D classification and 3D classification. Finally, 229,726 particles were refined and subjected to 3D refinement at 3.33 Å resolution in an overall structure.

Model building and refinement

The predicted 3D model of AtphyA and ZmphyA1 from Alpha Fold[5] was used as the initial template. The model of AtphyA and ZmphyA1 was built into the map of the dimeric phyA-Pr complex by using Coot[6]. For AtphyA-Pr, model of apo-AtphyA was fitted as rigid bodies followed by manual adjustment and rebuilding in Coot. The PDB file of PΦB was generated from the cryo-EM structure corresponding to PDB file 7rzw.The automatic model refinement was performed by real-space refinement (phenix.real_space_refine) in Phenix[7] and manual adjustment in COOT. Figure preparation and structure analysis were performed with PyMOL (pymol.org) and UCSF ChimeraX[8].

ADDITIONAL DISCUSSION

In this study, we solved cryo-EM structures of apo-AtphyA, AtphyA-Pr and ZmphyA1-Pr, which revealed similar asymmetric homodimers. Structural comparison of apo-AtphyA and AtphyA-Pr showed that PΦB binding induces reorganization of the elements composing the PΦB-binding cradle. The nearly identical structures of ZmphyA1-Pr and AtphyA-Pr agree with their spectral characteristics[9], further strengthening the conclusion that conserved mechanisms are involved in phyA-mediated light sensing in monocots and dicots. Intriguingly, a putative Class I nuclear localization signal (NLS) is included in the 380s loop (residues N344-K361) in phyA-GAF[10]. A chimeric phyB protein with its GAF substituted by phyA-GAF is localized to the nucleus in darkness[11]. Interestingly, replacement of the C-terminal region of the chimaera with the corresponding part of phyA cancels the nuclear localization phenotype. Hence, the NLS in the 380s loop is thought to be sequestered by the C-terminus of phyA. However, the 380s loop in the full-length structure of phyA containing the PAS2 and HKRD domains is flexible (Supplementary

information, Fig. S8a), possibly wobbling at the opposite side of the PSM-PAS2 platform relative to HKRDs, making it difficult to explain the sequestration of the NLS by the C-terminal region. A previous study suggested that phyA is an intrinsic kinase with the nPAS-GAF-PHY module required for kinase activity and a putative ATP-binding pocket formed by the α1 and α7 helices of the PHY domain[12]. However, tight packing of two helices of the PHY domain in the putative pocket leaves little room to accommodate an ATP molecule (Supplementary information, Fig. S8e).

Despite their conserved PΦB-binding mechanisms, AtphyA-Pr and AtphyB-Pr differ in their N-terminal PSMs. The PSM of AtphyA-Pr is stretched by a less twisted tongue from the PHY domain, giving rise to a slightly wider PSM-PAS2 platform. Structural alignment showed that PΦB binding induces no striking conformational changes in AtphyA-Pr and that the bound PΦB is completely buried in a phy protein, suggesting that the local conformational changes in the PΦB binding site may be sufficient for signalling. PHY tongue contact with PΦB is essential for Pr to Pfr photoconversion[13-15]. A PHY-deleted version of AtphyB generated a normal Pr conformer but could not be photoconverted to the Pfr state[16], consistent with the role of PHY in stabilizing Pfr conformers. Specific residue mutations in the tongue of the PHY domain altered the Pr/Pfr interconversion and the photosensitivity of phys[16-19]. In addition, the combination of the PHY tongue with the approaching modulator loop in PAS2 was speculated to compose a photostate sensing interface[20]. Given that the PHY tongue of phys is transformed into a helical structure to stabilize their Pfr conformers, as observed in bacterial relatives[21,22], the structural divergences in the tongue of phyA-Pr and phyB-Pr are expected to affect the transformation efficiency and consequently their photosensitivities.

When phyA controls physiological processes, it typically mediates VLFR when etiolated seedlings are still under the soil surface and another photoresponse, FR-HIR, in a far-red light-enriched environment, such as under leaf canopies. Hence, phyA is well known as a highly sensitized far-red light photoreceptor. It is also of evolutionary significance for the emergence of phyA in early angiosperms, as the properties of phyA provide adaptive advantages in a shady environment dominated by gymnosperms and ferns. Given that the structure of phyA-Pr offers adequate biological relevance to its high photosensitivity, how can the role of phyA as a far-red light photoreceptor be explained? For both phyA and phyB in the equilibrium of Pr and Pfr interconversion, red light effectively induces most Pfr conformers, and far-red light merely induces traces of Pfr conformers (approximately 2% of the total phy proteins). Hence, a less stable Pr

conformer for an easier and quicker transformation of Pr to Pfr and a stronger Pfr conformer to counterbalance the trace amounts seem to be essential for phyA as a far-red light photoreceptor. The structure of phyA-Pr correlates with the former; however, the structure of its Pfr conformer is urgently needed to address this long-term challenge.

1 Zheng, S. Q. *et al.* MotionCor2: anisotropic correction of beam-induced motion for improved cryo-electron microscopy. *Nat Methods* **14**, 331-332 (2017).

2 Zhang, K. Gctf: Real-time CTF determination and correction. *Journal of Structural Biology* **193**, 1-12 (2016).

3 Kimanius, D., Dong, L. Y., Sharov, G., Nakane, T. & Scheres, S. H. W. New tools for automated cryo-EM single-particle analysis in RELION-4.0. *Biochem J* **478**, 4169-4185 (2021).

4 A. Punjani, J. L. Rubinstein, D. J. Fleet, M. A. Brubaker, cryoSPARC: algorithms for rapid unsupervised cryo-EM structure determination. *Nat Methods* **14**, 290-296 (2017).

5 Jumper, J. *et al.* Highly accurate protein structure prediction with AlphaFold. *Nature* **596**, 583-+ (2021).

6 P. Emsley, B. Lohkamp, W. G. Scott, K. Cowtan, Features and development of Coot. *Acta Crystallogr D Biol Crystallogr* **66**, 486-501 (2010).

7 Afonine, P. V. *et al.* Real-space refinement in PHENIX for cryo-EM and crystallography. *Acta Crystallographica Section D-Structural Biology* **74**, 531-544 (2018).

8 Pettersen, E. F. *et al.* UCSF ChimeraX: Structure visualization for researchers, educators, and developers. *Protein Sci.* **30**, 70-82 (2021).

9 Burgie, E. S. *et al.* Differing biophysical properties underpin the unique signaling potentials within the plant phytochrome photoreceptor families. *P Natl Acad Sci USA* **118** (2021).

10 Nagano, S. *et al.* Structural insights into photoactivation and signalling in plant phytochromes. *Nat Plants* **6** (2020).

11 Oka, Y. *et al.* Arabidopsis Phytochrome A Is Modularly Structured to Integrate the Multiple Features That Are Required for a Highly Sensitized Phytochrome. *Plant Cell* **24**, 2949-2962 (2012).

12 Shin, A. Y. *et al.* Evidence that phytochrome functions as a protein kinase in plant light signalling. *Nat Commun* **7** (2016).

13 Matsushita, T., Mochizuki, N. & Nagatani, A. Dimers of the N-terminal domain of phytochrome B are functional in the nucleus. *Nature* **424**, 571-574 (2003).

14 Oka, Y. *et al.* Functional analysis of a 450-amino acid N-terminal fragment of phytochrome B in Arabidopsis. *Plant Cell* **16**, 2104-2116 (2004).

15 Viczian, A. *et al.* A Short Amino-Terminal Part of Arabidopsis Phytochrome A Induces Constitutive Photomorphogenic Response. *Molecular Plant* **5**, 629-641 (2012).

16 Burgie, E. S., Bussell, A. N., Walker, J. M., Dubiel, K. & Vierstra, R. D. Crystal structure of the photosensing module from a red/far-red light-absorbing plant phytochrome. *P Natl Acad Sci USA* **111**, 10179-10184 (2014).

17 Kretsch, T., Poppe, C. & Schafer, E. A new type of mutation in the plant photoreceptor phytochrome B causes loss of photoreversibility and an extremely enhanced light sensitivity. *Plant J* **22**, 177-186 (2000).

18 Maloof, J. N. *et al.* Natural variation in light sensitivity of Arabidopsis. *Nat Genet* **29**, 441-446 (2001).

19 Anders, K., Daminelli-Widany, G., Mroginski, M. A., von Stetten, D. & Essen, L. O. Structure of the Cyanobacterial Phytochrome 2 Photosensor Implies a Tryptophan Switch for Phytochrome Signaling. *J Biol Chem* **288**, 35714-35725 (2013).

20 Li, H., Burgie, E. S., Gannam, Z. T. K., Li, H. L. & Vierstra, R. D. Plant phytochrome B is an asymmetric dimer with unique signalling potential. *Nature* **604**, 127-+ (2022).

21 Takala, H. *et al.* Signal amplification and transduction in phytochrome photosensors. *Nature* **509**, 245-+ (2014).

22 Burgie, E. S., Zhang, J. R. & Vierstra, R. D. Crystal Structure of Deinococcus Phytochrome in the Photoactivated State Reveals a Cascade of Structural Rearrangements during Photoconversion. *Structure* **24**, 448-457 (2016).

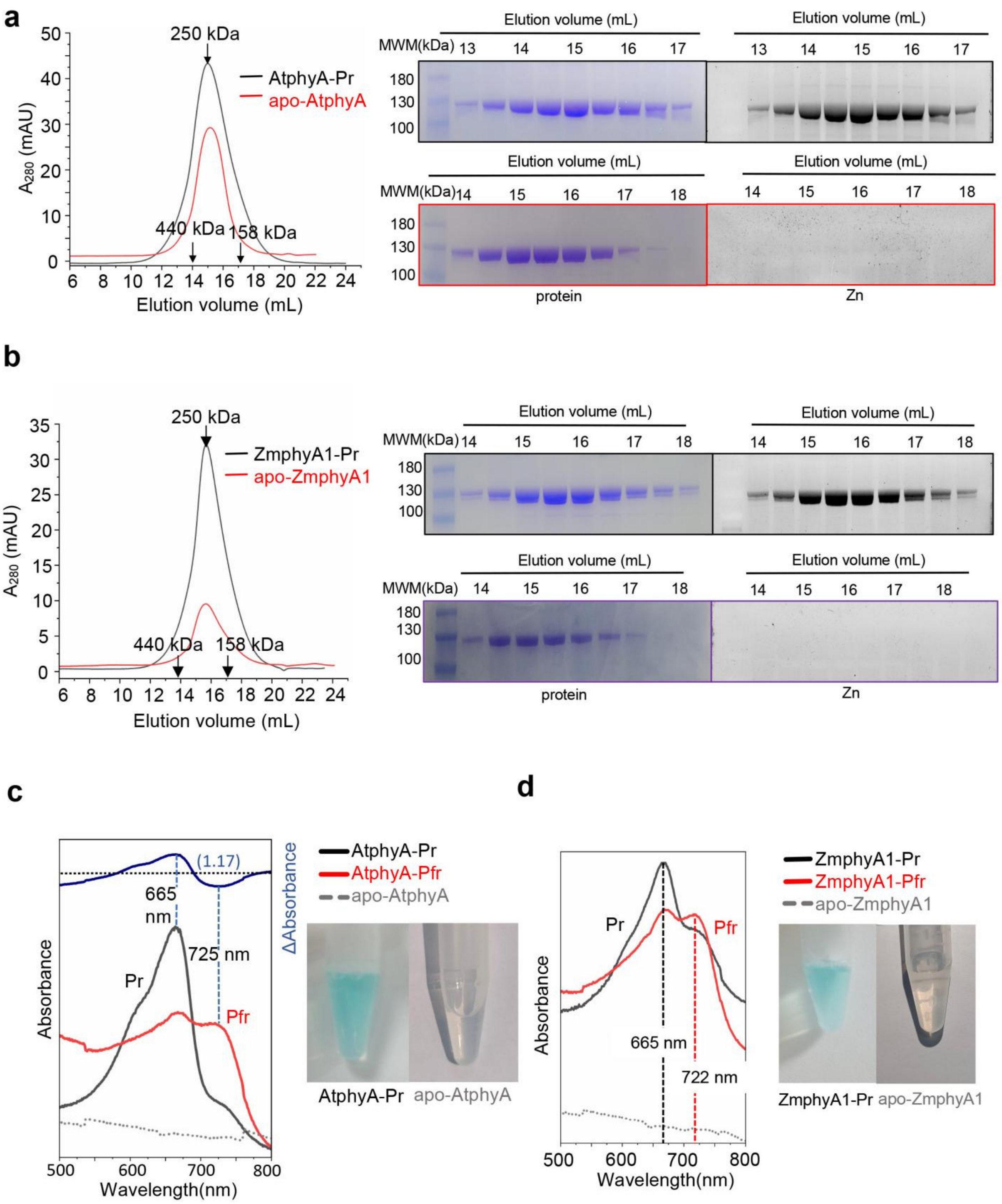
a
250 kDa
AtphyA-Pr
apo-AtphyA
A280 (mAU)
440 kDa
158 kDa
Elution volume (mL)
MWM(kDa)
protein
Zn
b
250 kDa
ZmphyA1-Pr
apo-ZmphyA1
440 kDa
158 kDa
Elution volume (mL)
protein
Zn
c
(1.17)
665 nm
725 nm
ΔAbsorbance
Absorbance
Pr
Pfr
Wavelength(nm)
AtphyA-Pr
AtphyA-Pfr
apo-AtphyA
AtphyA-Pr apo-AtphyA
d
Pr
Pfr
665 nm
722 nm
Absorbance
Wavelength(nm)
ZmphyA1-Pr
ZmphyA1-Pfr
apo-ZmphyA1
ZmphyA1-Pr apo-ZmphyA1

Fig. S1 Purification and absorbance spectra of phyA proteins.

a. Left: Gel filtration profiles of the AtphyA-Pr (black) and apo-AtphyA (red) proteins. Positions of standard molecular weights are indicated by the black arrows. right: Peak fractions in the left were visualized by SDS-PAGE followed by Coomassie blue staining (left) and zinc-induced fluorescence assay (right).

b. Left: Gel filtration profiles of ZmphyA1-Pr (black) and apo-ZmphyA1 (red) proteins. Positions of standard molecular weights are indicated by black arrows. Right: Peak fractions in the top were visualized by SDS-PAGE followed by Coomassie blue staining (left) and zinc-induced fluorescence assay (right).

c. Left: UV-vis absorbance spectra of apo-AtphyA (dashed black line), AtphyA proteins in Pr (black line), and after saturating irradiation with red light (red line, Pfr increased). The spectral change ratio (SCR) at 725 nm is 1.17. Right: a cyan color of AtphyA-Pr and the colorless apo-AtphyA.

d. Left: UV-vis absorbance spectra collected from apo-ZmphyA1 (dashed black line), ZmphyA1 proteins in Pr (black line), and after saturating irradiation with red light (red line, Pfr increased). Right: a cyan color of ZmphyA1-Pr and the colorless apo-ZmphyA1.

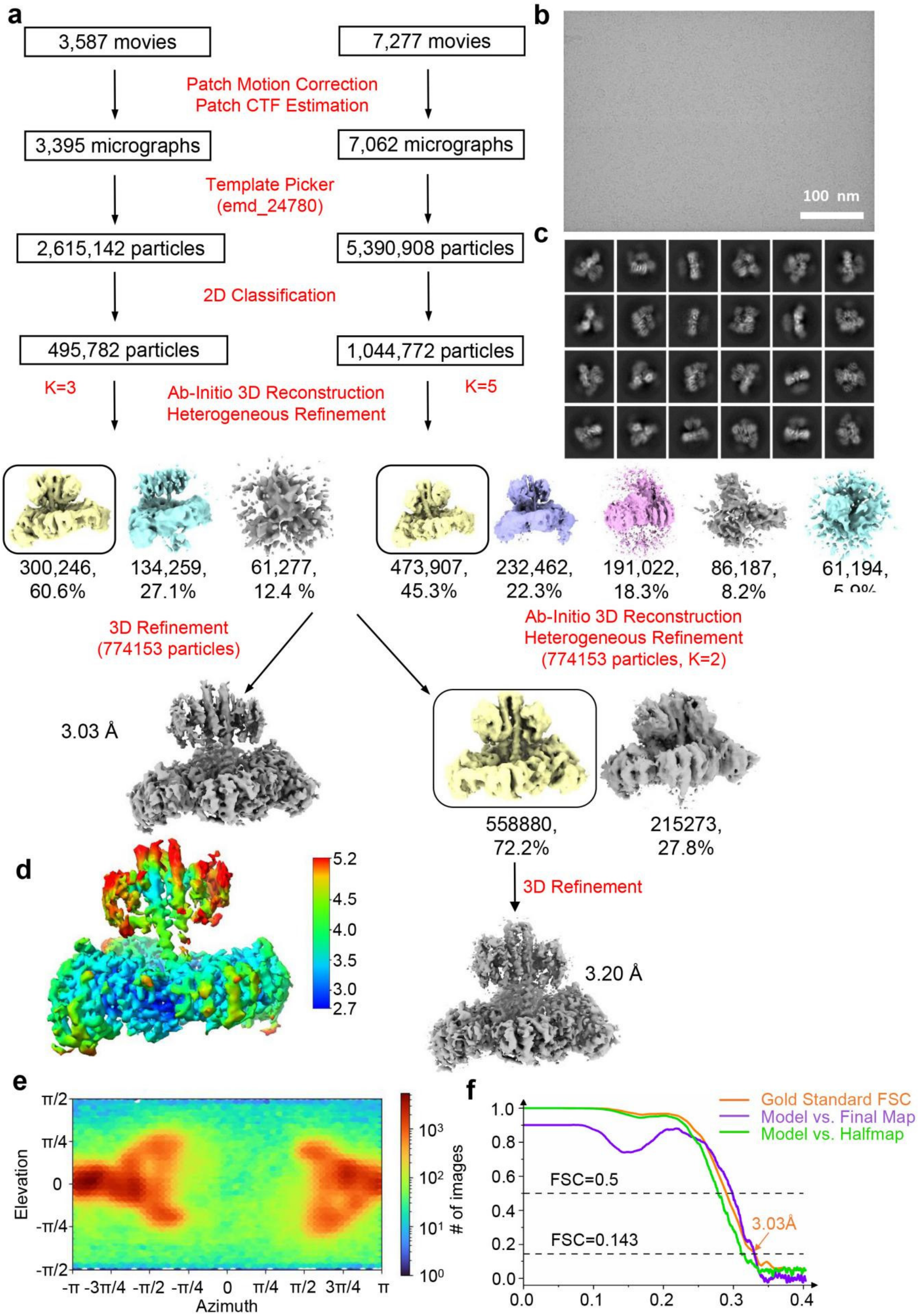
a
3,587 movies
7,277 movies
Patch Motion Correction
Patch CTF Estimation
3,395 micrographs
7,062 micrographs
Template Picker
(emd_24780)
2,615,142 particles
5,390,908 particles
2D Classification
495,782 particles
1,044,772 particles
K=3
Ab-Initio 3D Reconstruction
Heterogeneous Refinement
K=5
300,246, 60.6%
134,259, 27.1%
61,277, 12.4 %
473,907, 45.3%
232,462, 22.3%
191,022, 18.3%
86,187, 8.2%
61,194,
3D Refinement
(774153 particles)
Ab-Initio 3D Reconstruction
Heterogeneous Refinement
(774153 particles, K=2)
3.03 Å
558880, 72.2%
215273, 27.8%
3D Refinement
3.20 Å
b
100 nm
c
d
5.2
4.5
4.0
3.5
3.0
2.7
e
π/2
π/4
0
-π/4
-π/2
Elevation
-π -3π/4 -π/2 -π/4 0 π/4 π/2 3π/4 π
Azimuth
10^3
10^2
10^1
10^0
# of images
f
Gold Standard FSC
Model vs. Final Map
Model vs. Halfmap
FSC=0.5
FSC=0.143
3.03Å
1.0
0.8
0.6
0.4
0.2
0.0
0.0 0.1 0.2 0.3 0.4

Fig. S2 Workflow and resolution estimation for the cryo-EM map of AtphyA-Pr.

a. Flowchart for data processing of the cryo-EM micrographs of AtphyA-Pr.

b. A representative cryo-EM micrograph of AtphyA-Pr.

c. Representative views of 2D class averages of the AtphyA-Pr.

d. Color-coded local resolution of the final 3D cryo-EM map of the AtphyA-Pr.

e. Eulerian angle distribution of raw particle images used in the final 3D refinement.

f. Gold-standard Fourier shell correlation (FSC) curve for the final 3D refinement and the validation of the atomic model by correlation curves comparing the model to the final and half map.

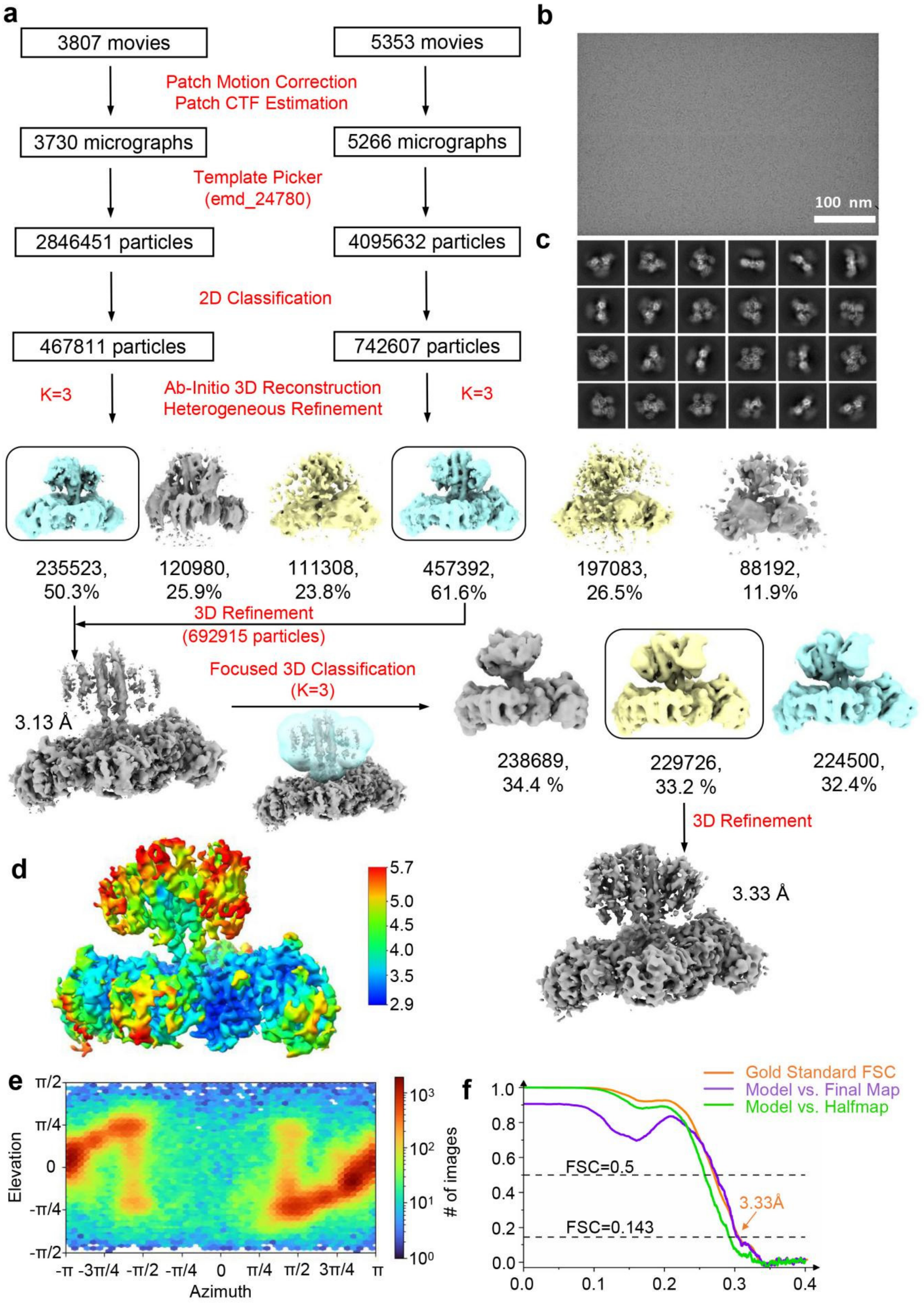
a
3807 movies
5353 movies
Patch Motion Correction
Patch CTF Estimation
3730 micrographs
5266 micrographs
Template Picker
(emd_24780)
2846451 particles
4095632 particles
2D Classification
467811 particles
742607 particles
K=3
Ab-Initio 3D Reconstruction
Heterogeneous Refinement
K=3
235523, 50.3%
120980, 25.9%
111308, 23.8%
457392, 61.6%
197083, 26.5%
88192, 11.9%
3D Refinement
(692915 particles)
3.13 Å
Focused 3D Classification
(K=3)
238689, 34.4 %
229726, 33.2 %
224500, 32.4%
3D Refinement
3.33 Å
b
100 nm
c
d
5.7
5.0
4.5
4.0
3.5
2.9
e
Elevation
Azimuth
# of images
f
Gold Standard FSC
Model vs. Final Map
Model vs. Halfmap
FSC=0.5
FSC=0.143
3.33Å

Fig. S3 Workflow and resolution estimation for the cryo-EM map of ZmphyA1-Pr.

a. Flowchart for data processing of the cryo-EM micrographs of ZmphyA1-Pr.
b. A representative cryo-EM micrograph of ZmphyA1-Pr.
c. Representative views of 2D class averages of the ZmphyA1-Pr.
d. Color-coded local resolution of the final 3D cryo-EM map of the ZmphyA1-Pr.
e. Eulerian angle distribution of raw particle images used in the final 3D refinement.
f. Gold-standard Fourier shell correlation (FSC) curve for the final 3D refinement and the validation of the atomic model by correlation curves comparing the model to the final and half map.

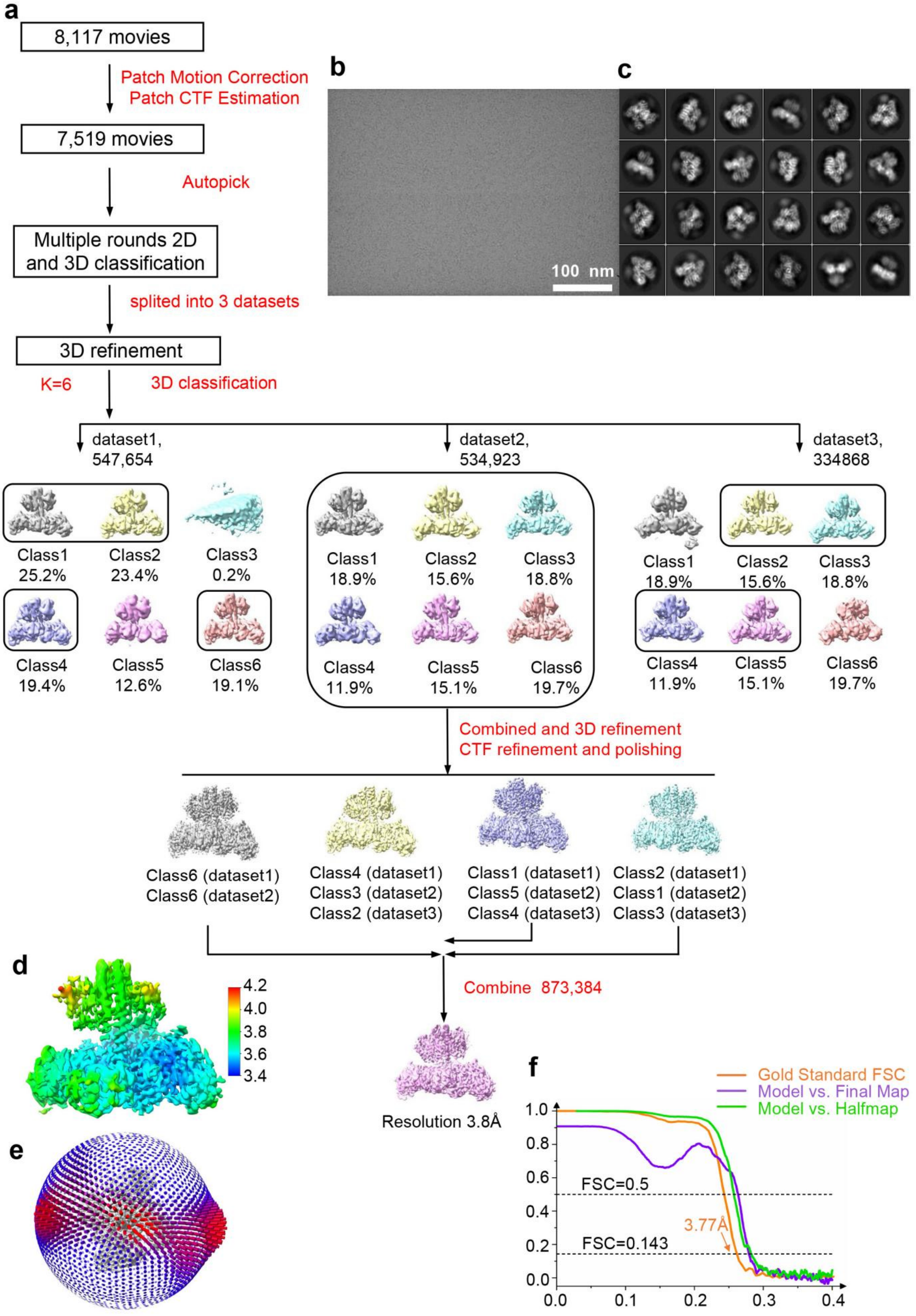
a
8,117 movies
Patch Motion Correction
Patch CTF Estimation
7,519 movies
Autopick
Multiple rounds 2D and 3D classification
splited into 3 datasets
3D refinement
K=6
3D classification
dataset1, 547,654
dataset2, 534,923
dataset3, 334868
Class1 25.2%
Class2 23.4%
Class3 0.2%
Class4 19.4%
Class5 12.6%
Class6 19.1%
Class1 18.9%
Class2 15.6%
Class3 18.8%
Class4 11.9%
Class5 15.1%
Class6 19.7%
Class1 18.9%
Class2 15.6%
Class3 18.8%
Class4 11.9%
Class5 15.1%
Class6 19.7%
Combined and 3D refinement
CTF refinement and polishing
Class6 (dataset1)
Class6 (dataset2)
Class4 (dataset1)
Class3 (dataset2)
Class2 (dataset3)
Class1 (dataset1)
Class5 (dataset2)
Class4 (dataset3)
Class2 (dataset1)
Class1 (dataset2)
Class3 (dataset3)
Combine 873,384
Resolution 3.8Å
b
100 nm
c
d
4.2
4.0
3.8
3.6
3.4
e
f
Gold Standard FSC
Model vs. Final Map
Model vs. Halfmap
FSC=0.5
FSC=0.143
3.77Å
1.0
0.8
0.6
0.4
0.2
0.0
0.0
0.1
0.2
0.3
0.4

Fig. S4 Workflow and resolution estimation for the cryo-EM map of apo-AtphyA.

a. Flowchart for data processing of the cryo-EM micrographs of apo-AtphyA.

b. A representative cryo-EM micrograph of apo-AtphyA.

c. Representative views of 2D class averages of the apo-AtphyA.

d. Color-coded local resolution of the final 3D cryo-EM map of the apo-AtphyA.

e. Eulerian angle distribution of raw particle images used in the final 3D refinement.

f. Gold-standard Fourier shell correlation (FSC) curve for the final 3D refinement and the validation of the atomic model by correlation curves comparing the model to the final and half map.

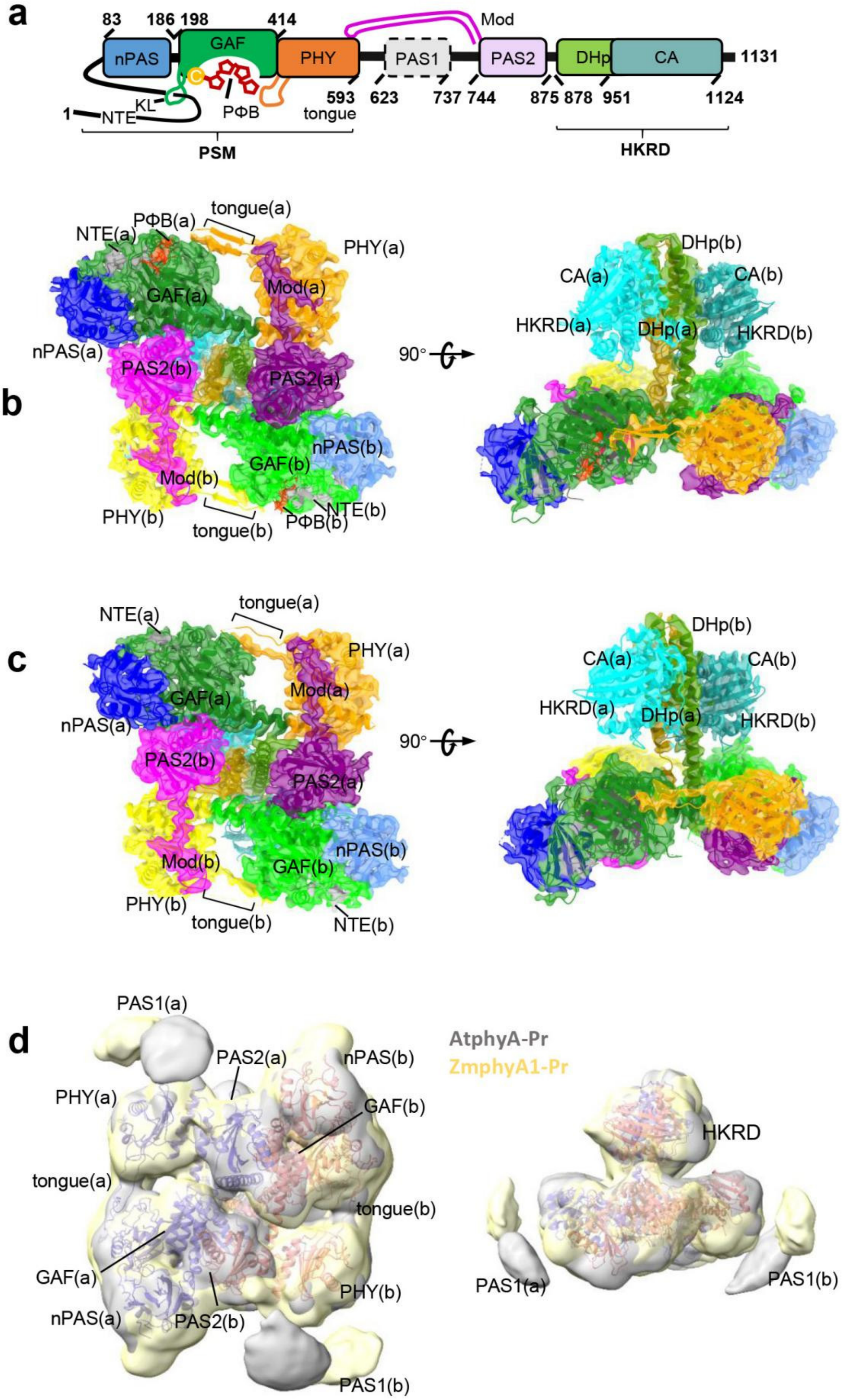
a
83
186 198
414
Mod
nPAS
GAF
PHY
PAS1
PAS2
DHp
CA
1131
593
tongue
623
737 744
875 878
951
1124
1
NTE
KL
PΦB
PSM
HKRD
b
NTE(a)
PΦB(a)
tongue(a)
PHY(a)
Mod(a)
GAF(a)
nPAS(a)
PAS2(b)
PAS2(a)
GAF(b)
nPAS(b)
Mod(b)
PHY(b)
tongue(b)
NTE(b)
PΦB(b)
90°
DHp(b)
CA(a)
CA(b)
HKRD(a)
DHp(a)
HKRD(b)
c
NTE(a)
tongue(a)
PHY(a)
Mod(a)
GAF(a)
nPAS(a)
PAS2(b)
PAS2(a)
nPAS(b)
Mod(b)
GAF(b)
PHY(b)
tongue(b)
NTE(b)
90°
DHp(b)
CA(a)
CA(b)
HKRD(a)
DHp(a)
HKRD(b)
d
PAS1(a)
AtphyA-Pr
ZmphyA1-Pr
PAS2(a)
nPAS(b)
PHY(a)
GAF(b)
HKRD
tongue(a)
tongue(b)
GAF(a)
PHY(b)
nPAS(a)
PAS2(b)
PAS1(a)
PAS1(b)
PAS1(b)

Fig. S5 Overall structures of phyA-related asymmetric dimers.

a. Color-coded domain architecture of full-length ZmphyA1. The N-terminal PSM containing NTE, nPAS, GAF and its knot lasso (KL), PHY and its tongue, the internal PAS1-PAS2 along with the modulator loop (Mod), the C-terminal HKRD comprising DHp and CA subdomains are shown. The boundaries are indicated by the numbers.
b. Cryo-EM map of ZmphyA1-Pr superposed with atomic model in cartoon shown in three orientations.
c. Cryo-EM map of apo-AtphyA superposed with atomic model in cartoon shown in three orientations.
d. The low-pass filtered cryo-EM map of AtphyA-Pr is fitted into that of ZmphyA1-Pr to compare the packing of their PAS1s. The fitted model in cartoon is AtphyA-Pr.

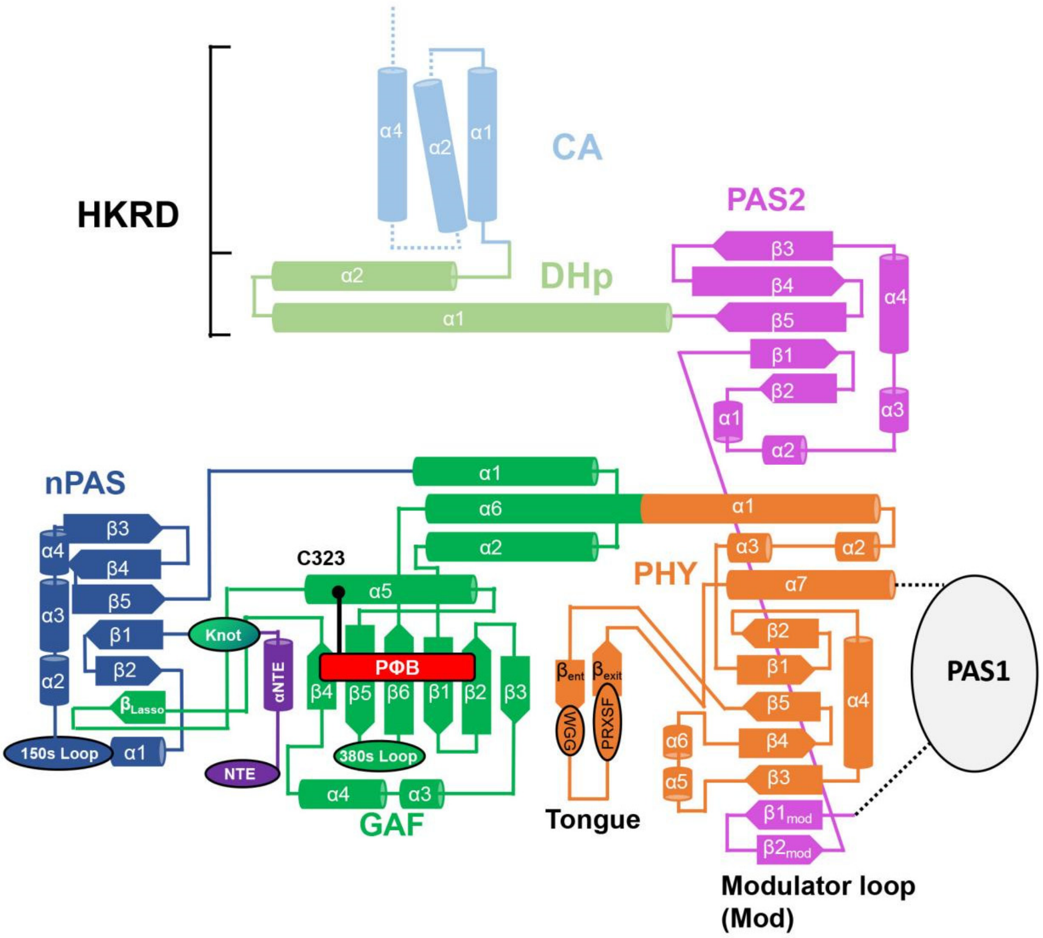

Fig. S6 Topology of AtphyA-Pr generated from its structure model.

Topological schematic of AtphyA-Pr. The secondary structural elements of the N-terminal PSM containing NTE, nPAS, GAF and its knot lasso (KL), PHY and tongue, the internal PAS1-PAS2 along with the modulator loop (Mod), the C-terminal HKRD comprising DHp and CA subdomains are shown. Only the α1, α2, α4 helices in CA subdomains are well defined in our cryo-EM structure.

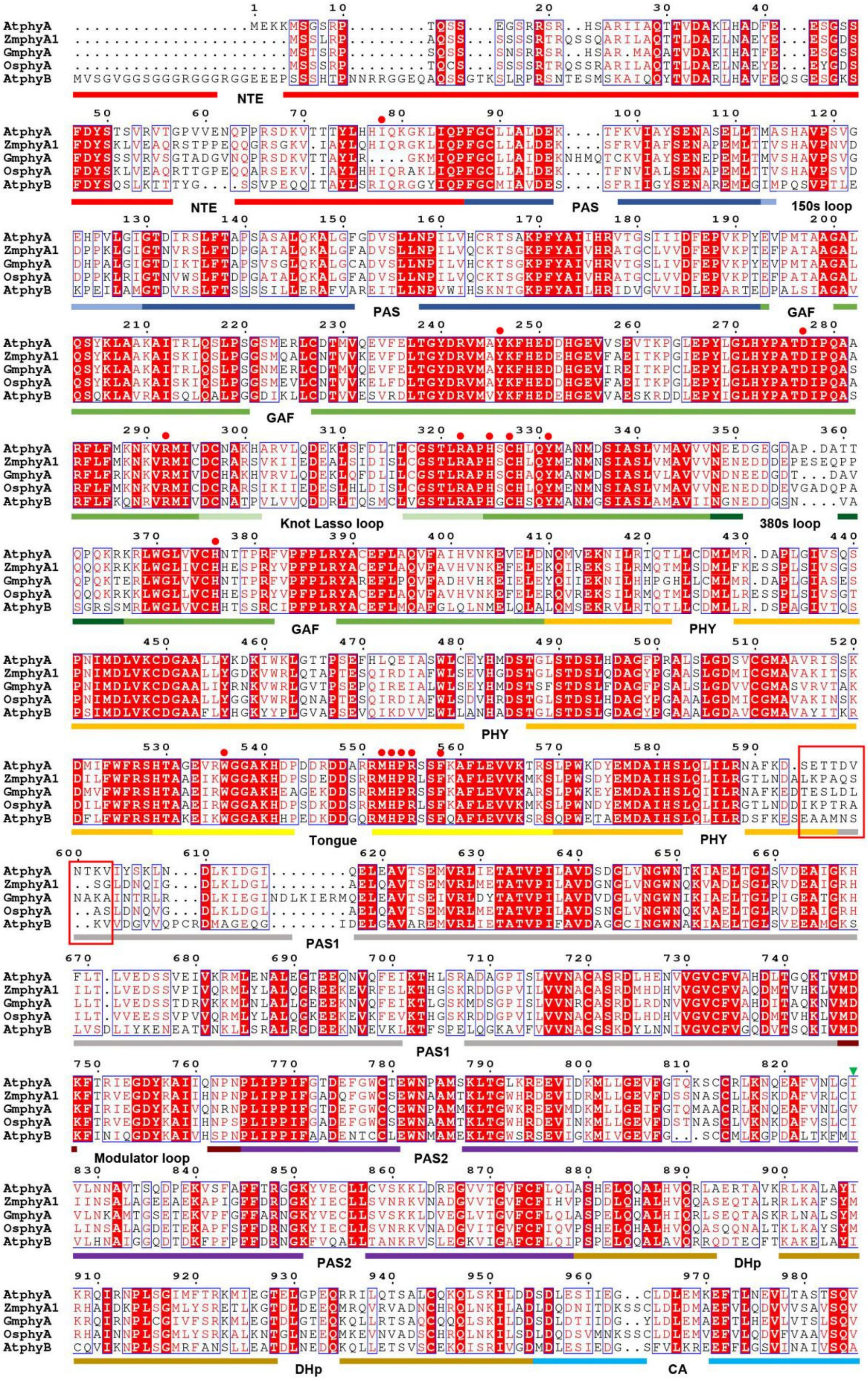
AtphyA
ZmphyA1
GmphyA
OsphyA
AtphyB
NTE
PAS
150s loop
GAF
Knot Lasso loop
380s loop
PHY
Tongue
PAS1
Modulator loop
PAS2
DHp
CA

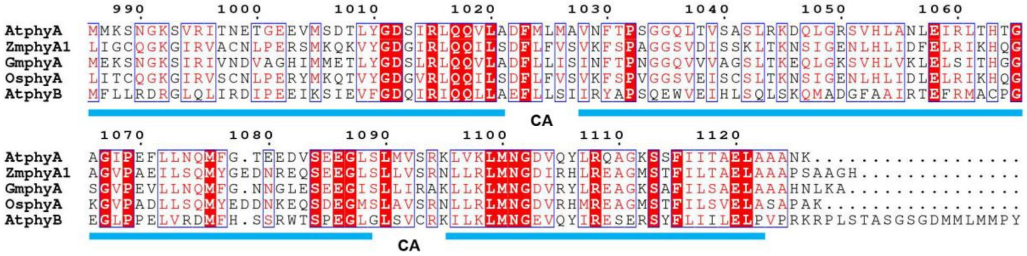

Fig. S7 Amino acid sequence alignment of phyA and phyB from different species.

The positions of the NTE, nPAS, GAF, PHY, PAS1, PAS2, and DHp, CA subdomains of the HKRD are located by the red, blue, green, orange, gray, purple, brown and cyan bars, respectively. The 150s and 380s loops, the knot lasso, the tongue and the modulator loops are located by light blue, light green, dark green, yellow, and dark red bars, respectively. The red box indicates residues linking PHY and PAS1 domains. The solid red circles indicate the residues involved in PΦB-binding. At: *Arabidopsis thaliana*; Zm: *Zea mays*; Gm: *Glycine max*; Os: *Oryza sativa*.

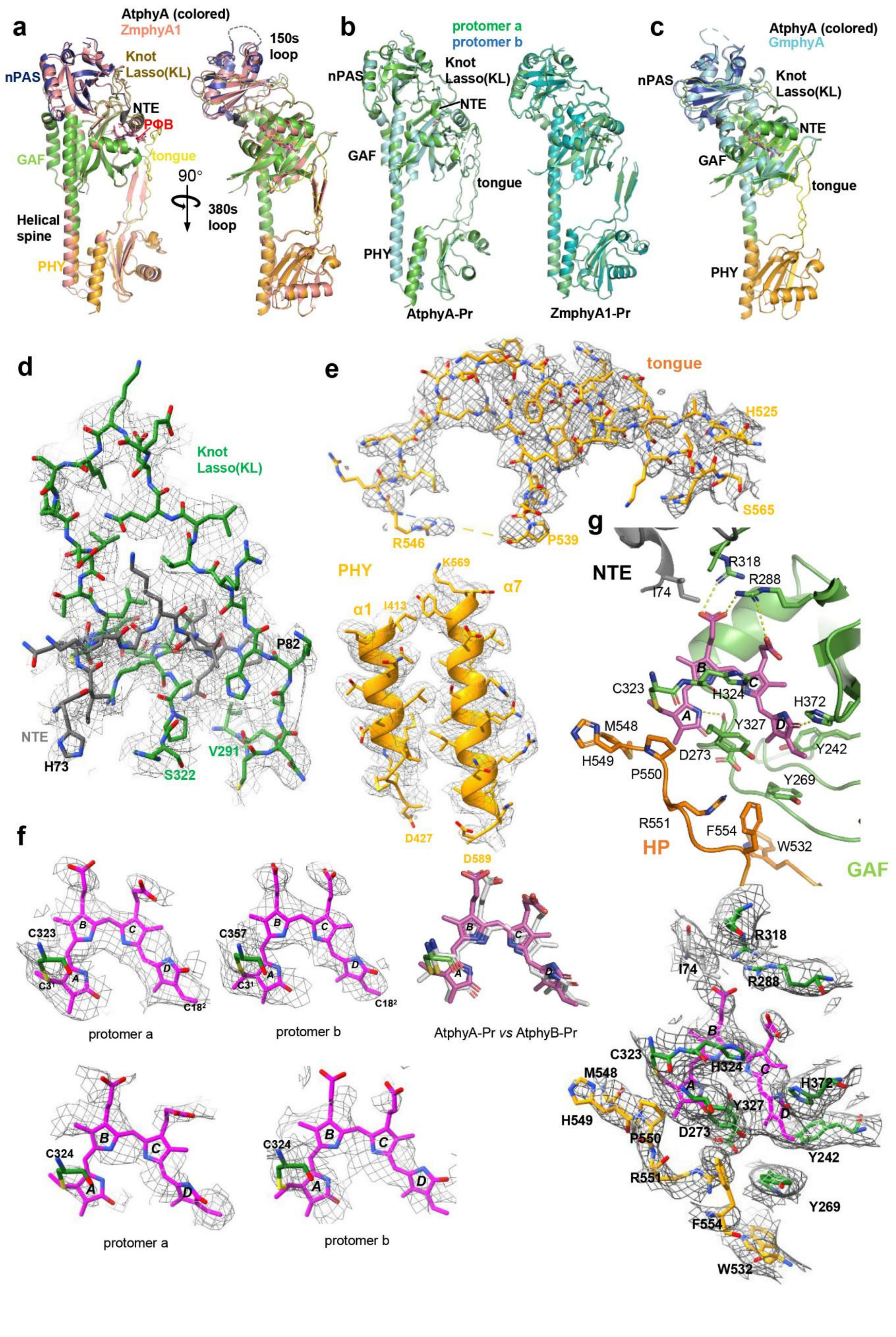
a
AtphyA (colored)
ZmphyA1
150s loop
nPAS
Knot Lasso(KL)
NTE
PΦB
GAF
tongue
90°
380s loop
Helical spine
PHY
b
protomer a
protomer b
nPAS
Knot Lasso(KL)
NTE
GAF
tongue
PHY
AtphyA-Pr
ZmphyA1-Pr
c
AtphyA (colored)
GmphyA
nPAS
Knot Lasso(KL)
NTE
GAF
tongue
PHY
d
Knot Lasso(KL)
P82
NTE
H73
S322
V291
e
tongue
H525
S565
R546
P539
PHY
K569
α1
α7
I413
D427
D589
f
C323
C3¹
C18²
protomer a
C357
C3¹
C18²
protomer b
AtphyA-Pr vs AtphyB-Pr
C324
protomer a
C324
protomer b
g
NTE
I74
R318
R288
C323
H324
H372
M548
Y327
H549
D273
Y242
P550
Y269
R551
F554
W532
HP
GAF
R318
I74
R288
C323
H324
M548
H372
Y327
H549
P550
D273
Y242
R551
Y269
F554
W532

Fig. S8 Structure of the N-terminal PSM in phyA-Pr and the PΦB-binding mechanism.

a. The PSM of AtphyA-Pr (color-coded) is similar to that of ZmphyA1-Pr (salmon). The helical spine connecting the GAF and PHY domains, the 150s loop in nPAS and 380s loop in GAF are indicated. Residues from D540 to A545 in the PHY tongue are missing in the structure of AtphyA-Pr and residues including the tongue tip cannot be modelled in the structure of ZmphyA1-Pr.

b. The PSMs in two protomers of AtphyA-Pr and ZmphyA1-Pr are identical.

c. The PSM of AtphyA-Pr (color-coded) is well aligned with the nPAS-GAF fragment of GmphyA-Pr (cyan, PDB code: 6TC7).

d. The cyro-EM densities (grey mesh) well define the residues (shown in stick) modelled in the NTE and knot lasso (KL) of GAF domain in AtphyA-Pr.

e. The cyro-EM densities (grey mesh) well define the residues (shown in stick) modelled in the tongue (top), α1 and α7 helices (bottom) of PHY domain in AtphyA-Pr.

f. Top: The cyro-EM densities (grey mesh) of PΦB (shown in stick) modelled in protomer a (left) and b (middle) of AtphyA-Pr. Shown at right is the alignment of PΦBs in AtphyA-Pr and AtphyB-Pr. Bottom: The cyro-EM densities (grey mesh) define the PΦB (shown in stick) modelled in protomer a (left) and b (right) of ZmphyA1-Pr.

g. Top: The PΦB-binding pocket in AtphyA-Pr and the interacting residues from the NTE, GAF and PHY tongue are shown in stick. The dashed yellow lines indicate hydrogen bonds. Bottom: The cyro-EM densities (grey mesh) well define the residues (shown in stick) involved in PΦB-binding shown in top.

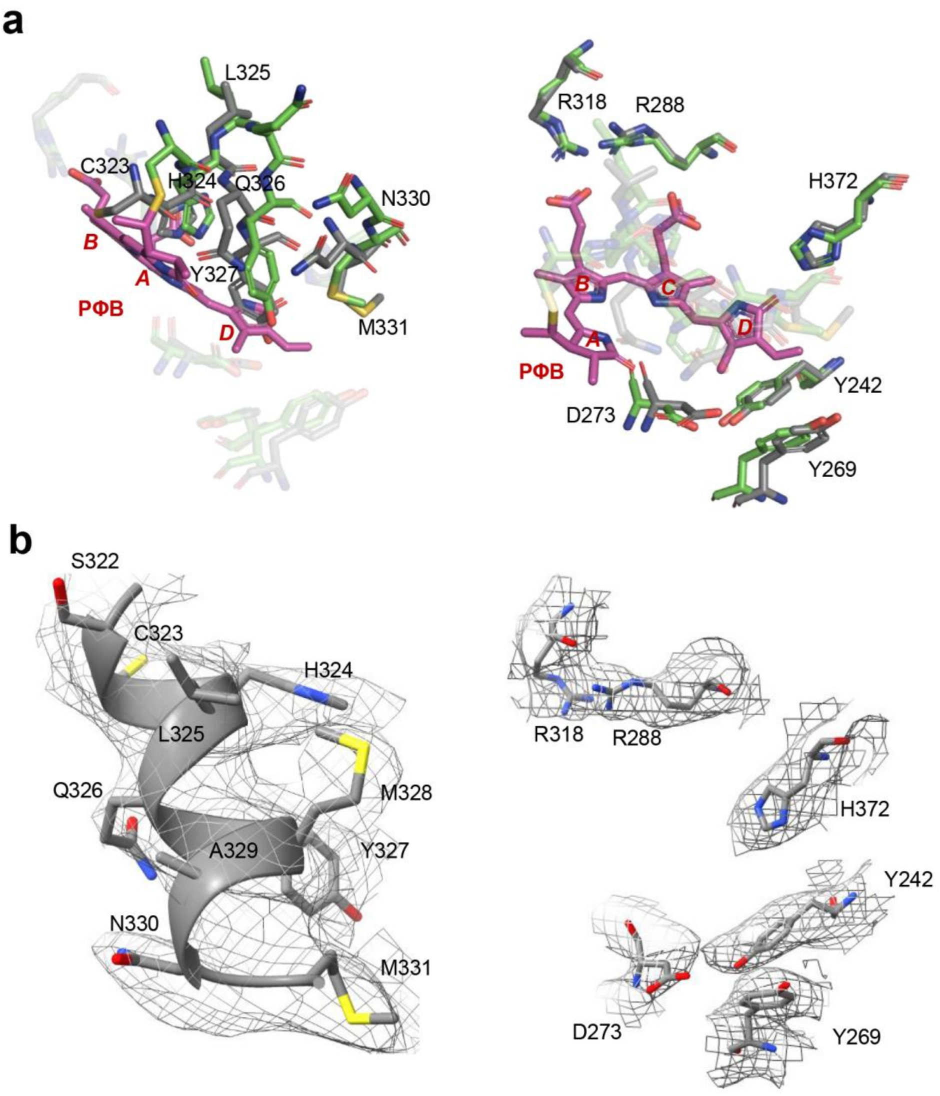

Fig. S9 Comparison of the PΦB-binding pocket in AtphyA-Pr and apo-AtphyA.

a. The key residues (shown in stick) involved in PΦB binding in the GAF helix-α5 (left) and the other parts of its pocket (right) of AtphyA-Pr (green) and apo-AtphyA (grey) are superposed.

b. The cyro-EM densities (grey mesh) well define the residues (shown in stick) involved in PΦB-binding in the GAF helix-α5 (left) and the other parts of its pocket (right) of apo-AtphyA.

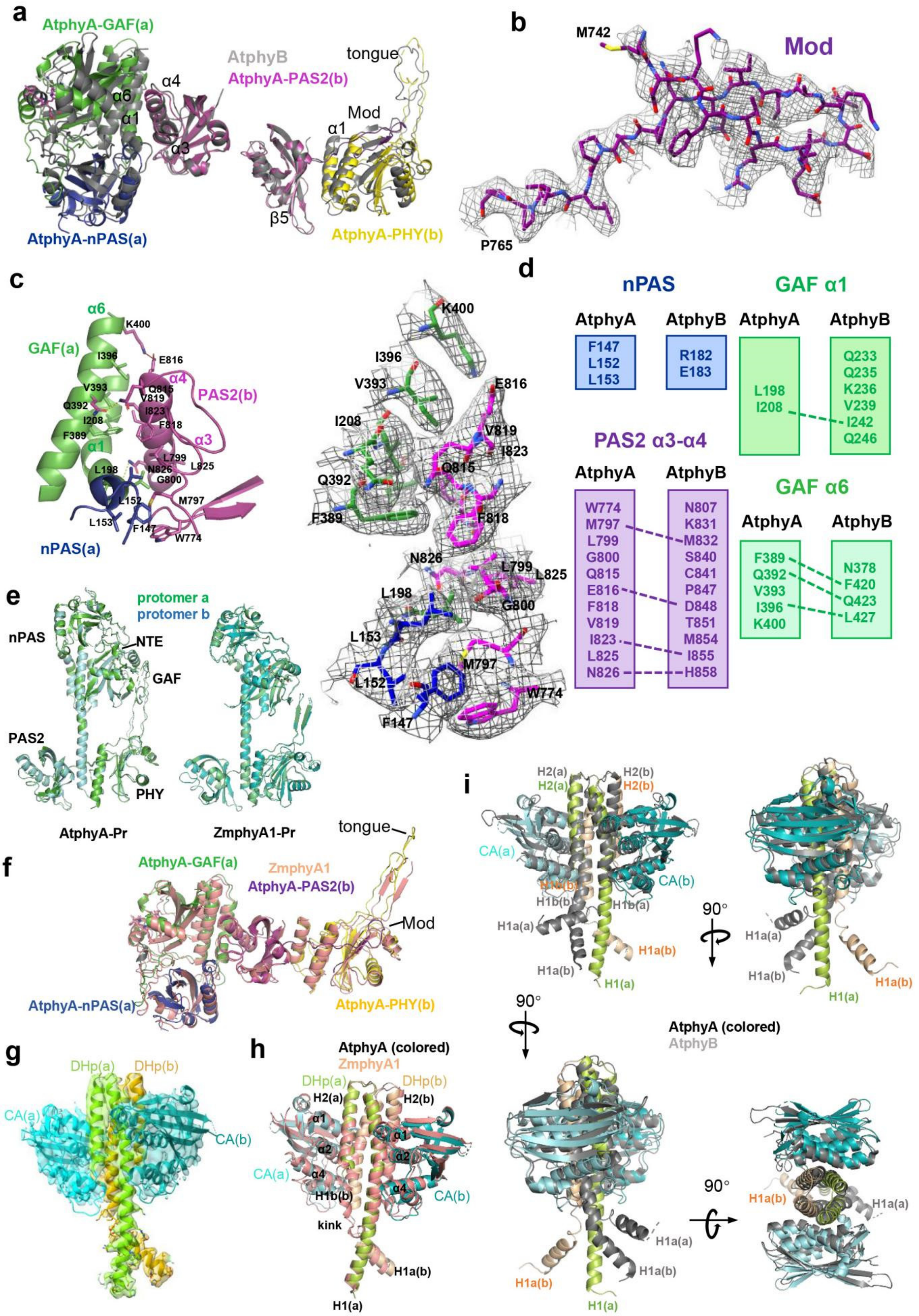
a
AtphyA-GAF(a)
AtphyB
AtphyA-PAS2(b)
tongue
α4
α6
α1
α3
Mod
α1
β5
AtphyA-nPAS(a)
AtphyA-PHY(b)
b
M742
Mod
P765
c
α6
K400
GAF(a)
I396
E816
V393
α4
Q815
V819
PAS2(b)
Q392
I823
I208
F818
F389
α1
α3
L198
L799
N826
L825
G800
L152
M797
L153
F147
W774
nPAS(a)
d
nPAS
AtphyA
AtphyB
F147
L152
L153
R182
E183
GAF α1
AtphyA
AtphyB
L198
I208
Q233
Q235
K236
V239
I242
Q246
PAS2 α3-α4
AtphyA
AtphyB
W774
M797
L799
G800
Q815
E816
F818
V819
I823
L825
N826
N807
K831
M832
S840
C841
P847
D848
T851
M854
I855
H858
GAF α6
AtphyA
AtphyB
F389
Q392
V393
I396
K400
N378
F420
Q423
L427
e
protomer a
protomer b
nPAS
NTE
GAF
PAS2
PHY
AtphyA-Pr
ZmphyA1-Pr
f
tongue
AtphyA-GAF(a)
ZmphyA1
AtphyA-PAS2(b)
Mod
AtphyA-nPAS(a)
AtphyA-PHY(b)
g
DHp(a)
DHp(b)
CA(a)
CA(b)
h
AtphyA (colored)
ZmphyA1
DHp(a)
DHp(b)
H2(a)
H2(b)
α1
α1
α2
α2
CA(a)
α4
α4
CA(b)
H1b(b)
kink
H1a(b)
H1(a)
i
H2(a)
H2(a)
H2(b)
H2(b)
CA(a)
CA(b)
H1b(b)
H1b(a)
H1a(a)
H1a(b)
H1a(b)
H1(a)
90°
H1a(a)
H1a(b)
H1(a)
H1a(b)
90°
AtphyA (colored)
AtphyB
H1a(a)
H1a(b)
H1(a)
H1a(b)
90°
H1a(b)
H1a(a)

Fig. S10 Structure of the C-terminal region in phyA-Pr and the dimerization mechanism.

a. The PAS2-(nPAS-GAF) interprotomer interface (left) and PAS2-PHY intraprotomer interface (right) in AtphyA-Pr (color-coded) and AtphyB-Pr (grey, PDB code: 7RZW). The helices and strands composing the interfaces, the modulator loop (Mod) in PAS2 and tongue in PHY are indicated. In the PAS2-(nPAS-GAF) interprotomer interface, α3 and α4 of PAS2 contact α1 and α6 of GAF and a small turn in the nPAS of the other protomer primarily through hydrophobic interactions. In the PAS2-PHY intraprotomer interface, PAS2 packs against its adjacent PHY mainly via the interactions between the β5 strand in the PAS2 and the α1 helix in the PHY.

b. The cyro-EM densities (grey mesh) well define the residues (shown in stick) modelled in the modulator loop (Mod) of PAS2 domain in AtphyA-Pr.

c. Left: Interactions in the PAS2-(nPAS-GAF) interprotomer interface in AtphyA-Pr. The interacting residues are shown in stick. The dashed yellow and red lines indicate hydrogen bonds and electrostatic interactions, respectively. Right: The cyro-EM densities (grey mesh) well define the residues (shown in stick) involved in PAS2-(nPAS-GAF) interprotomer interface shown in left.

d. Comparison of critical residues involved in PAS2-(nPAS-GAF) interprotomer interface between AtphyA-Pr and AtphyB-Pr. Dashed lines indicate the conserved amino acids.

e. The PSM-PAS2 modules in two protomers of AtphyA-Pr and ZmphyA1-Pr are identical.

f. The PAS2-(nPAS-GAF) interprotomer interface and PAS2-PHY intraprotomer interface are conserved in AtphyA-Pr (color-coded) and ZmphyA1-Pr (salmon).

g. The cyro-EM densities (colored surface) well define the secondary structure elements (shown in cartoon) containing the H1 and H2 helices of DHp subdomain, and α1, α2, α4 helices in CA subdomains of HKRD in AtphyA-Pr.

h. Conserved “head to head” asymmetric homodimers of HKRDs in AtphyA-Pr (color-coded) and ZmphyA1-Pr (salmon). A kink occurs to H1 of protomer b, resulting in the H1a(b) and H1b(b).

i. Asymmetric HKRD homodimers of AtphyA-Pr (color-coded) and AtphyB-Pr (grey, PDB code: 7RZW) are shown in four orientations. Both the two H1 helices in AtphyB-Pr possess kinks. The four-helix bundle containing H1b and H2 helices in DHp subdomains and its tightly packing CA subdomains are well superposed in the two phys. The two H1a helices are not

symmetrically equivalent in the two protomers, and also cannot align to each other between AtphyA-Pr and AtphyB-Pr.

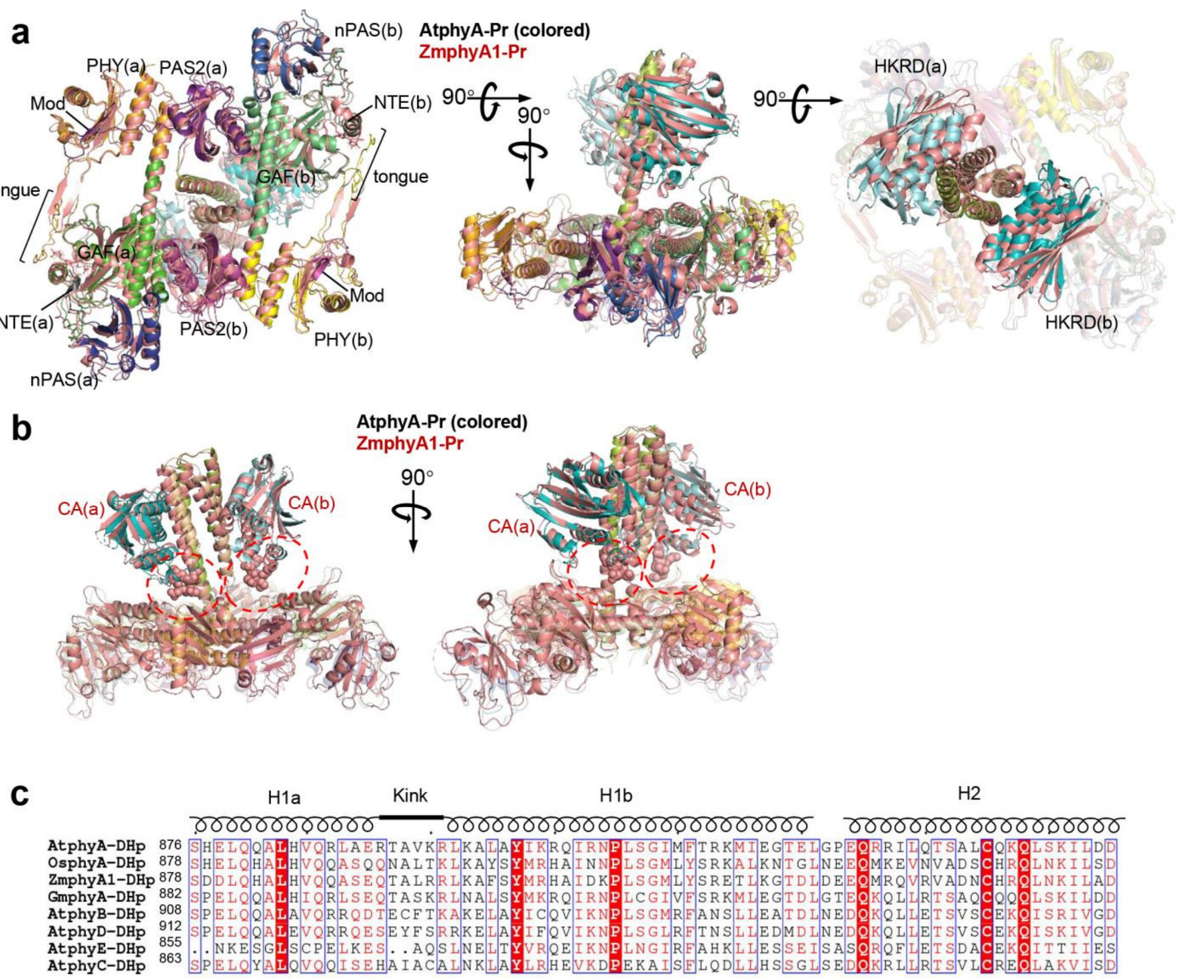

Fig. S11 Structures of phyA-Pr are conserved in monocot and dicot.

a. Structural comparison of AtphyA-Pr (color-coded) and ZmphyA1-Pr (salmon) shown in three orientations. The two structures are well superposed and their tilting angles of HKRD dimers are same.

b. Structural comparison of AtphyA-Pr (color-coded) and ZmphyA1-Pr (salmon) shown in another two orientations to highlight no contacts between HKRDs and PSMs in ZmphyA1-Pr.

c. Sequence alignment of DHp subdomains in different phys indicates the sequences of kink regions are not conserved. At: *Arabidopsis thaliana*; Os: *Oryza sativa*; Zm: *Zea mays*; Gm: *Glycine max*.

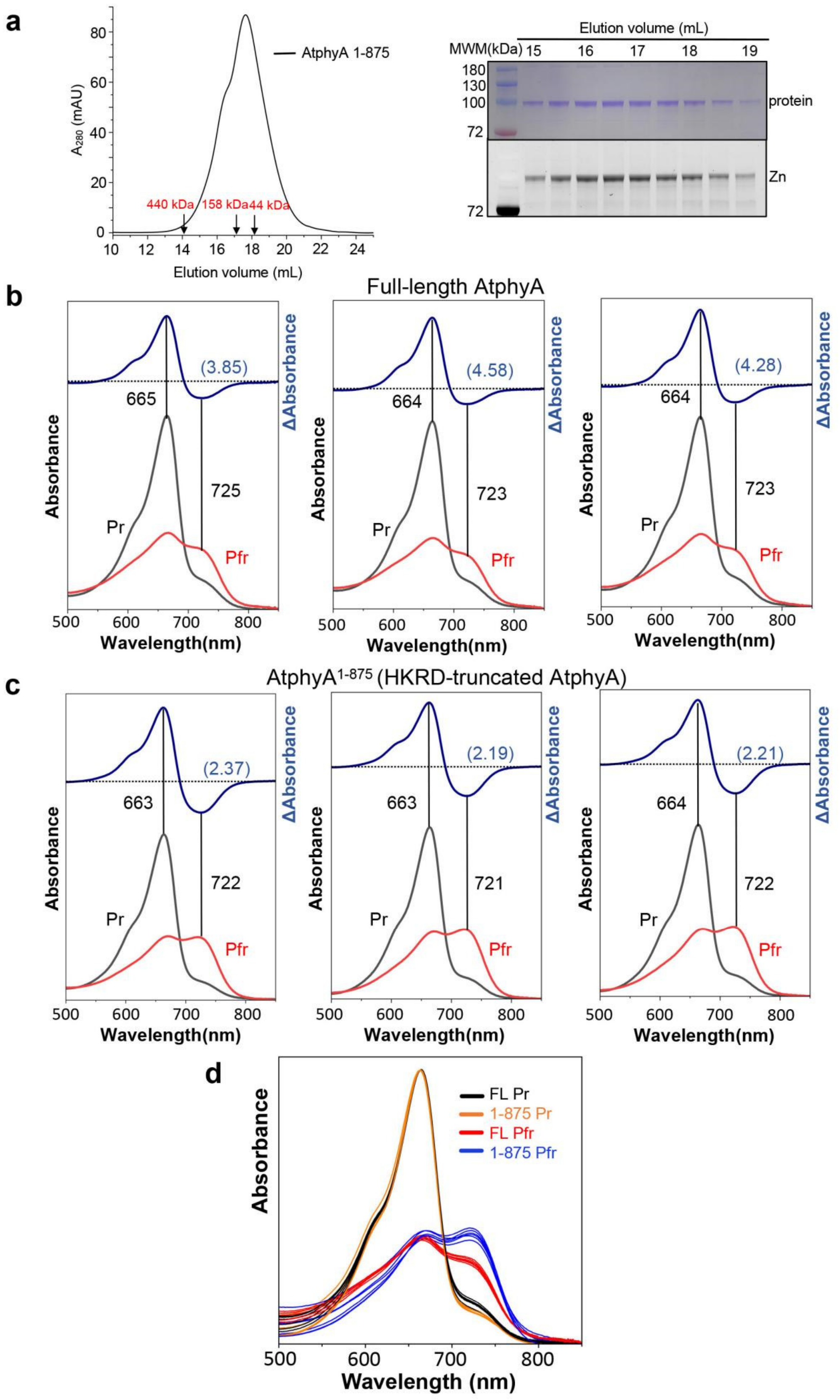
a
AtphyA 1-875
A280 (mAU)
Elution volume (mL)
440 kDa
158 kDa
44 kDa
Elution volume (mL)
MWM(kDa)
15
16
17
18
19
180
130
100
72
protein
Zn
b
Full-length AtphyA
ΔAbsorbance
Absorbance
(3.85)
665
725
(4.58)
664
723
(4.28)
664
723
Pr
Pfr
Wavelength(nm)
c
AtphyA1-875 (HKRD-truncated AtphyA)
(2.37)
663
722
(2.19)
663
721
(2.21)
664
722
d
FL Pr
1-875 Pr
FL Pfr
1-875 Pfr
Absorbance
Wavelength (nm)

Fig. S12 Purification and spectral characteristics of phyA-ΔHKRD proteins.

a. Left: Gel filtration profiles of phyA-ΔHKRD proteins. Positions of standard molecular weights are indicated by the black arrows. Right: Peak fractions in the left were visualized by SDS-PAGE followed by Coomassie blue staining (left) and zinc-induced fluorescence assay (right).
b. UV-vis absorbance spectra of full-length AtphyA proteins in Pr (black line), after saturating irradiation with red light (red line, Pfr increased). SCR is indicated in parenthesis. Spectra were measured by three technical replicates.
c. UV-vis absorbance spectra of HKRD-truncated AtphyA proteins in Pr (black line), after saturating irradiation with red light (red line, Pfr increased). SCR is indicated in parenthesis. Spectra were measured by three technical replicates.
d. The normalized UV-vis absorbance spectra shown in **b** and **c**.

Table S1. Cryo-EM statistics and model refinement for apo-AtphyA, AtphyA-Pr and ZmphyA1-Pr

	apo-AtphyA	AtphyA-Pr	ZmphyA1-Pr
PDB ID	8ISI	8ISJ	8ISK
EMDB ID	35691	35692	35693
**Data collection and processing**			
Microscope		Thermo Scientific Krios G4	
Detector		Gatan K3 Cameras	
Magnification		105,000	
Pixel size (Å)		0.85	
Total electron dose (e^-/Å2)		50	
Exposure rate (e^-/pixel/sec)		15	
Defocus range (μm)	-1.0 — -2.0	-1.0 — -2.0	-1.0 — -2.0
Movies collected	8,117	10,864	9,160
Micrographs used	7,519	10,457	8,996
**Reconstruction**			
Software	RELION-4.0.0	cryoSAPRC-v4.1.2	cryoSAPRC-v4.1.2
Total extraced particles	6,300,932	8,006,050	6,942,083
Number of particles used for 3D reconstruction	2,601,275	1,540,554	1,210,418
Number of particles used for refinement	873,384	774,153	229,726
Symmetry	C1	C1	C1
Map Resolution (Å) FSC 0.143 after post-processing	3.8	3.0	3.3
Map Resolution range (Å)	3.4-4.2	2.7-5.2	2.9-5.7
Map sharpening B-factor (Å^2)	-150	-128.6	-111.2
**Refinement**			
Software		Coot-0.9.8.7+phenix.real_space_refine in the PHENIX-v1.20.1 package	
Model Resolution (Å) FSC 0.5	3.8	3.4	3.7
Model composition			
Number of chains	2	2	2
Number of atoms	13,097 (Hydrogens: 0)	13,534 (Hydrogens: 0)	13,277 (Hydrogens: 0)
Number of protein residues	1,675	1,716	1,693
Number of ligands	0	2	2
R.M.S deviations			
Bonds lengths (Å)	0.003 (0)	0.003 (0)	0.003 (0)
Bonds angles (°)	0.603 (0)	0.695 (0)	0.639 (0)
ADP (B-factors)			
Protein	59.51	96.21	115.98
Ligand	---	28.06	136.83
Model vs. Map			
CC (mask)	0.79	0.80	0.78
CC (box)	0.79	0.75	0.70
Mean CC for ligands	---	0.64	0.54
**Validation**			
MolProbity overall score	1.69	1.79	1.77
All-atom clashscore	7.70	8.92	9.65
Rotamer outliers (%)	0.07	0.20	0.00
C-beta deviations	0.00	0.00	0.00
Cis/Twisted peptides (%)	0.0/0.0	0.0/0.0	0.0/0.0
Ramachandran plot statistics			
Preferred (%)	96.12	95.51	96.11
Allowed (%)	3.88	4.43	3.89
Outlier (%)	0.00	0.06	0.00

Table S2. Summary of model building of apo-AtphyA, AtphyA-Pr and ZmphyA1-Pr

		Calculated length (aa)	Well-modeled segments (residue #)	Modeled segments with uncertainty (residue #)	unassigned segments (residue #)
**apo-AtphyA**	protomer a	1122	PSM (75-109, 121-348, 361-535, 556-590); PAS2 (743-873); HKRD-DHp (874-950)	HKRD-CA (951-1041, 1051-1119)	PSM (1-74, 110-120, 349-360, 536-555); PAS1 (591-742); HKRD (1042-1050, 1120-1122)
	protomer b	1122	PSM (75-110, 120-347, 360-534, 562-590); PAS2 (743-873); HKRD-DHp (874-950)	HKRD-CA (951-1039, 1052-1119)	PSM (1-74, 111-119, 348-359, 535-561); PAS1 (591-742); HKRD (1040-1051, 1120-1122)
**AtphyA-Pr**	protomer a	1122	PSM (73-110, 121-347, 361-539, 546-589); PAS2 (742-873); HKRD-DHp (874-950)	HKRD-CA (951-1041, 1050-1120)	PSM (1-72, 111-120, 348-360, 540-545); PAS1 (590-741); HKRD (1042-1049, 1121-1122)
	protomer b	1122	PSM (73-110, 120-347, 361-539, 546-588); PAS2 (742-873); HKRD-DHp (874-950)	HKRD-CA (951-1040, 1050-1119)	PSM (1-72, 111-119, 348-360, 540-545); PAS1 (589-741); HKRD (1041-1049, 1120-1122)
**ZmphyA1-Pr**	protomer a	1131	PSM (73-110, 123-347, 363-537, 559-591); PAS2 (745-876); HKRD-DHp (877-954)	HKRD-CA (955-1045, 1053-1125)	PSM (1-72, 111-122, 348-362, 538-558); PAS1 (592-744); HKRD (1046-1052, 1126-1131)
	protomer b	1131	PSM (72-110, 123-347, 362-537, 559-591); PAS2 (743-876); HKRD-DHp (877-954)	HKRD-CA (955-1045, 1054-1125)	PSM (1-71, 111-122, 348-361, 538-558); PAS1 (592-742); HKRD (1046-1053, 1126-1131)

aa: amino acid
